# Seasonal variations in carbon, nitrogen and phosphorus concentrations and C:N:P stoichiometry in different organs of a *Larix principis-rupprechtii* Mayr. plantation in the Qinling Mountains, China

**DOI:** 10.1371/journal.pone.0185163

**Published:** 2017-09-22

**Authors:** Hailiang Li, M. James C. Crabbe, Fuli Xu, Weiling Wang, Lihui Ma, Ruilong Niu, Xing Gao, Xingxing Li, Pei Zhang, Xin Ma, Haikui Chen

**Affiliations:** 1 College of Natural Resources and Environment, Northwest A&F University, Yangling, Shaanxi, P. R. China; 2 Wolfson College, Oxford University, Oxford, United Kingdom; 3 College of Life Science, Northwest A&F University, Yangling, Shaanxi, P. R. China; 4 Institute of Soil and Water Conservation, Northwest A&F University, Yangling, Shaanxi, P. R. China; 5 College of Biological Science and Engineering, Beifang University of Nationalities, Yinchuan, Ningxia, P. R. China; Tennessee State University, UNITED STATES

## Abstract

Understanding how concentrations of elements and their stoichiometry change with plant growth and age is critical for predicting plant community responses to environmental change. We used long-term field experiments to explore how the leaf, stem and root carbon (C), nitrogen (N) and phosphorous (P) concentrations and their stoichiometry changed with growth and stand age in a *L*. *principis-rupprechtii* Mayr. plantation from 2012–2015 in the Qinling Mountains, China. Our results showed that the C, N and P concentrations and stoichiometric ratios in different tissues of larch stands were affected by stand age, organ type and sampling month and displayed multiple correlations with increased stand age in different growing seasons. Generally, leaf C and N concentrations were greatest in the fast-growing season, but leaf P concentrations were greatest in the early growing season. However, no clear seasonal tendencies in the stem and root C, N and P concentrations were observed with growth. In contrast to N and P, few differences were found in organ-specific C concentrations. Leaf N:P was greatest in the fast-growing season, while C:N and C:P were greatest in the late-growing season. No clear variations were observed in stem and root C:N, C:P and N:P throughout the entire growing season, but leaf N:P was less than 14, suggesting that the growth of larch stands was limited by N in our study region. Compared to global plant element concentrations and stoichiometry, the leaves of larch stands had higher C, P, C:N and C:P but lower N and N:P, and the roots had greater P and C:N but lower N, C:P and N:P. Our study provides baseline information for describing the changes in nutritional elements with plant growth, which will facilitates plantation forest management and restoration, and makes a valuable contribution to the global data pool on leaf nutrition and stoichiometry.

## Introduction

Carbon (C), nitrogen (N) and phosphorus (P) are three mineral nutrients that are essential for plant growth, and all are vital to the nutrient cycling and sustainable management of forest ecosystems. C forms the structural basis of a plant and fairly consistently constitutes 50% of the dry mass of a plant [1]. Plants play a critical role in the C cycle by C fixing through photosynthesis and releasing C through litter decomposition [2–4]. N is one of the most important nutritional elements; most plants are completely dependent on soil inorganic N because they lack symbiotic N fixation, and N promotes high leaf area index values and long photosynthesis durations [5–7]. P influences photosynthetic assimilation and biomass production in plants [8] and represents the main component of RNA, DNA and ATP. In plants, nutrient limitations can be recognized as an increase in growth (or biomass accumulation) in response to the addition of the limiting nutrient [4]. In forest ecosystems, N and P are most often the limiting nutrients [9], but C can also act as such [10].

The nutritional element concentrations in the different organs of a plant reflect nutrient uptake and utilization efficiency during plant growth and play a significant role in the maintenance of plant function and adaptations to the environment [11], and they change during the growing season in response to variations in the photosynthetic capabilities and nutritional requirements of different plant growth stages [12]. Changes in the absorption, accumulation and allocation of N and P with the growth stage have attracted special attention due to the application for implementing nutrient management practices in forest ecosystems. The patterns of N or P status in plant biomass, especially in the leaves, have been studied intensely, and the results have indicated that changes in the concentrations of nutritional elements are the function of photosynthetic C fixation and nutrient uptake and are generally observed in certain annual species or perennial species within a growing season or a year [13–15]. Thus, completely elucidating the relationship between nutritional element concentrations and the changes in plant tissues during the growing season in forest ecosystems would provide important insights for forest management and fertilization. Although previous studies have advanced our understanding of the patterns of nutritional elements in forest plants, there is no clear information about how C, N and P concentrations and C:N:P stoichiometric characteristics vary during the growing season and with stand age in plantation forest ecosystems.

Ecological stoichiometry is an effective method for studying multiple nutritional elements of different plant tissues in undergoing various ecological interactions, and it integrates different scales ranging from the individual to the ecosystem. Furthermore, it plays an important role in describing the responses of a plant to diverse environmental changes and is widely used to indicate the elemental compositions of living organisms. The C, N, and P of stoichiometry in tissues can affect decomposition, grazing susceptibility and species composition in forest ecosystems, so it can aid in identifying the flows of energy and element cycling across diverse ecosystems, which can advance our understanding of ecological dynamics and processes [16, 17]. Previous studies have indicated that the C:N, C:P and N:P ratios represent the capacity of plants for photosynthetic C fixation under N or P accumulation, and N:P ratios have been used as indicators to study nutrient limitations in adverse environments [18]. Elemental stoichiometry could be affected by certain environmental fluctuations (temperature, elevation, precipitation, and drought), plant physiological processes, plant phenotype, stand age and growing season [14, 15, 19], so elemental concentrations and C:N:P stoichiometric ratios can vary within different growth stages during plant ontogeny. Unfortunately, the effects of plant organ size within a life cycle are often not held constant, and increases in organ size may cause variations in elemental concentrations and stoichiometry. Therefore, the changes in the elemental concentrations and the C:N, C:P and N:P ratios in a plant become more complex with the longer lifespans and multiple growing periods of perennial species. Thus, this study aims to determine how the changes that occur during the growing season and increases in stand age can elucidate the adaptations of perennial plants to various environments.

The watershed between the Qinling Mountains and the Yellow River Basin of northern China was historically home to warm temperate evergreen broadleaf forests with a high biodiversity [20], but over the past century, the natural forests of the Qinling Mountains were logged and replaced by plantation forests [21]. The low-elevation forests of the foothills have been replaced by temperate deciduous trees, such as oak (*Quercus acutissima*, *Q*. *variabilis*), elm (*Ulmus* spp.), maple (*Acer* spp.) and ash (*Fraxinus* spp.) and coniferous forests are now observed at the middle elevations, and include *Larix principis-rupprechtii* Mayr. (*Prince Rupprecht's* Larch.), which is native to the mountainous regions of the provinces of Shaanxi, Henan and Hebei of northern China. *Larix principis-rupprechtii* Mayr. is a more appropriate tree species for forest plantations in northern China [22] and is used for plant recovery and greening [23].

In this study, we determined the C, N and P concentrations as well as the C:N, C:P and N:P ratios in different tissues of a *L*. *principis-rupprechtii* Mayr. plantation in different growing seasons from 2012–2015. In particular, we (1) investigated the seasonal variations in the concentrations, accumulation, allocation, and stoichiometric ratios of C, N and P in different plant tissues; (2) explored the relationships between the nutritional element concentrations, their stoichiometric ratios and the stand age (plant age); and (3) evaluated the effects of plant organs, sampling month (sampling time) and stand age on nutritional element concentrations and their stoichiometric ratios.

## Materials and methods

### Ethics statement

This study was authorized and facilitated by the forestry administration of Taibai County of Shaanxi Province, China, and all necessary permits were obtained. We confirm that our study caused no harm to the environment, that no human or animal subjects were used, that no endangered or protected species of plants were used, and that no other permits were required. We declare that the described study complied with the current laws of China.

### Site description

Our study was conducted in Taibai County of Baoji City, Shaanxi Province, China (N: 34°02’ 18.1”, E: 107°20’ 51.1”), from 2012–2015 (S1 Fig). The study site is characterized by a continental monsoon and mountain climate; the average annual temperature is 7.7°C; the annual precipitation is 1000 mm; and the average elevation is 1675 m. The soil type of the experimental field is Brown soil (Luvisol according to the FAO classification); the soil texture is sandy clay loam (SCL); and the basic characteristics of the initial soil (0–20 cm) are shown in Table 1. Larch plantations are widely distributed in this area and used as the main tree species for afforestation. Larch plantations account for a large area of the study region, approximately 128 ha, and most were young and mid-aged plantations at the time of the study.

**Table 1:** Initial soil (0–20 cm) properties of the study region.

Chemical characteristics	Nutrient content (g kg^-1^)				Nutrient classification	Description
	2012	2013	2014	2015		
Organic matter (SOM)	25.89	24.93	17.17	13.77	III (SOM content: 20–30 g kg^-1^)	Normal (III)
					V (SOM content: 10–20 g kg^-1^)	Very poor (V)
Total N (TN)	1.44	1.2	0.95	1.16	III (TN content: 1.0–1.5 g kg^-1^)	Normal (III)
					IV (TN content: 0.75–1.0 g kg^-1^)	Poor (IV)
Total P (TP)	1.53	1.51	1.52	0.67	I (TP content: > 1.0 g kg^-1^)	Very high (I)
					III (TP content: 0.6~0.8 g kg^-1^)	Normal (III)

The soil nutrient classification is based on the 1982 national standard classification for soil nutrient content, and Roman numerals represent different nutrition classifications. The nutrient classification standard is based on data from the Second China National Soil Survey [24].

### Experimental design and plant sampling

In April 2012, we established three replicate plots (20 × 20 m each) in our study region that were nearly identical in topography and the composition of the undergrowth vegetation. In this year, the larch plantations were 20 years-old (half-mature forests), and the mean forest density was 2500 individual trees per hectare. In the study area, larch plantation trees usually bud in late April (during late-April to early-May, the leaves of larch stands are too short and small to collect); grow quickly in July, August and early September; grow slowly in late September and early October; and drop all leaves by early November. Thus, the sampling times were scheduled for the middle of May, June, July, August, September, and October from 2012–2015. The growth stage of the larch plantations was divided into an early growing season (May-June), a fast-growing season (July-September) and a late-growing season (October).

The field experiment was arranged in a split-plot design with three replicates, and the size of each experimental plot was 20 × 20 m. Two of the three plots were adjacent to each other; an isolation strip consisting of two lines of trees was built; and a distance of at least 5 m was maintained between adjacent plots. The third plot was separated from the others by 0.5 km. The large experimental area and the number of replicates were sufficient to minimize location and inter-individual differences; the experiment was designed to ensure the validity of all statistical tests. Three 5 × 5 m replicate blocks were established in each plot, and sampling was random. When possible, samples were collected from the same large trees in each plot. To standardize sample collection, sun-exposed and fully expanded mature leaves were collected from five individual trees in each replication block in each plot (5-g samples (fresh mass) were obtained from each tree and placed in a paper envelope). Leaves were collected using a pole pruner (5 m), and one sample composed of at least five trees was placed in a separate bag. To avoid resampling and secondary resampling, we marked each sampled tree. All visible root tissues were collected from the soil (0–20 cm), and the roots of each sampled individual were carefully washed with tap water. From each individual in each plot, we collected an average of 10 root segments with lengths of approximately 10 cm. Stems were collected by an increment borer from stem diameters larger than 20 cm because this tool can split the stems of smaller trees and potentially result in mortality. Simultaneously, triplicate soil samples were randomly collected at a depth of 0–20 cm within the same quadrats in the middle of April from 2012–2015. All soil samples were air dried, passed through a 0.25-mm sieve to remove gravel and plant remnants, and ground into a fine powder for analysis.

### Measurements of plant and soil samples

The plant samples were thoroughly mixed to make a composite, stored in paper envelopes, oven dried at 105°C for 15 min, and then oven dried at 75°C for 24 h. The dry weights were determined, and dried samples were ground into a uniform powder that was fine enough to pass through a 1.0-mm sieve for analysis. The organic C (OC) in the plant samples and the soil was measured by the potassium dichromate/sulfuric acid mixture titration method [25]. The total N (TN) in the plant samples and the soil was measured using a semimicro-Kjeldahl method [26] with a Kjeldahl auto-analyzer (K9840 Kjeldahl Analyzer, Hanon, CHN). The total P (TP) in the plant samples and the soil was determined colorimetrically [27] with an ultraviolet spectrophotometer (UVmini-1240, Shimadzu, JPN). All data were expressed as the mass (mg g^-1^). The C, N, and P stoichiometric ratios of the different samples were calculated as OC vs. TN (C:N), OC vs. TP (C:P), and TN vs. TP (N:P).

### Statistical analysis

All data (S1 Table) were log_10_-transformed and Levene’s test was used to test for homogeneity of variance. The differences in the concentrations or stoichiometric ratios among growing seasons and plant organs were analyzed by one-way analysis of variance (ANOVA) and Tukey’s multiple comparison post hoc tests. All statistical analyses were performed with SPSS version 19.0 (SPSS Inc., USA). A general linear model (GLM) was also applied to evaluate the effects of sampling year (stand age), sampling month, plant organ (leaves, stems, and roots), and their interactions on the C, N, P concentrations and the C:N:P stoichiometry. The linear correlations among the C, N, and P concentrations and the C:N, C:P, and N:P ratios with stand age were determined using ordinary least squares regression (OLS) with the model *y* = a*x* + b. The cartograms were plotted using Sigma-Plot Suite V12.5 (Systat Software Inc., USA).

## Results

### Seasonal variations in the C, N and P concentrations of different organs of larch stands

Sampling month, sampling year (stand age), plant organ and their interactions significantly affected the C, N and P concentrations in different organs of larch stands from 2012–2015. GLM analysis showed that plant organ was the largest contributor to the variation in the N and P concentrations (90.38% and 90.14%, respectively), while the interaction (sampling year × plant organ × sampling month) determined the greatest amount of variation in the C concentration (25.08%), followed by plant organ (20.58%) (Table 2). For the leaf N and P concentrations, sampling month determined the greatest amount of variation (80.10% and 52.69%, respectively), while the leaf C concentration and the root C and P concentrations were influenced by sampling year (stand age) (42.92%, 33.70% and 63.05%, respectively). For the stem C, N, P concentrations and the root N concentrations, the largest contributor was the sampling year × sampling month interaction (Table 3).

**Table 2:** Results of general linear models (GLM) of the effects of sampling year (SY), sampling month (SM), and plant organ (PO) on C, N, P, and C:N:P stoichiometry.

Factors	d.f.	C		N		P		C:N		C:P		N:P	
		MS	SS%	MS	SS%	MS	SS%	MS	SS%	MS	SS%	MS	SS%
SY	3	0.019**	18.01	0.101**	0.47	0.663**	2.61	0.147**	0.69	0.540**	2.12	0.660**	15.17
PO	2	0.032**	20.58	28.901**	90.38	34.283**	90.14	28.646**	89.53	34.377**	90.09	1.235**	18.93
SM	5	0.002**	3.54	0.148**	1.16	0.031**	0.21	0.143**	1.12	0.034**	0.22	0.091**	3.49
SY×PO	6	0.003**	5.79	0.063**	0.59	0.083**	0.65	0.059**	0.56	0.092**	0.73	0.194**	8.90
SY×SM	15	0.003**	12.54	0.077**	1.82	0.120**	2.37	0.092**	2.15	0.122**	2.40	0.155**	17.86
PO×SM	10	0.001**	4.82	0.221**	3.46	0.082**	1.08	0.224**	3.51	0.094**	1.23	0.125**	9.57
SY×PO×SM	30	0.003*	25.08	0.027**	1.27	0.065**	2.56	0.033**	1.54	0.071**	2.80	0.086**	19.83
Residuals	144	>0.001	9.65	0.004	0.85	0.002	0.37	0.004	0.90	0.002	0.41	0.006	6.27

Type III sums of squares converted to percentages at each level. All data were log_10_-transformed before analysis; d.f. degree of freedom, MS: mean squares, SS%: percentage of the sum of squares explained (%); ns indicates not significant (P > 0.05);

* indicates statistically significant at the 0.05 significance level (* P < 0.05 and ** P < 0.001).

**Table 3:** Results of general linear models (GLM) of the effects of sampling year (SY) and sampling month (SM) on C, N, P, and C:N:P stoichiometry.

Plant organ	Factors	d.f.	C		N		P		C:N		C:P		N:P	
			MS	SS%	MS	SS%	MS	SS%	MS	SS%	MS	SS%	MS	SS%
Leaf	SY	3	0.007**	42.92	0.049**	5.39	0.065**	17.17	0.022**	2.39	0.034**	9.19	0.013**	2.99
	SM	5	0.002**	15.48	0.441**	80.10	0.120**	52.69	0.458**	82.95	0.139**	63.54	0.148**	55.90
	SY×SM	15	0.001**	25.21	0.025**	13.81	0.022**	29.16	0.025**	13.46	0.019**	25.62	0.035**	39.25
	Residuals	48	<0.001	16.38	<0.001	0.70	<0.001	0.97	0.001	1.20	<0.001	1.65	0.001	1.85
Stem	SY	3	0.011**	23.65	0.065*	9.63	0.469**	28.38	0.087**	11.53	0.419**	24.75	0.555**	24.07
	SM	5	0.001**	5.38	0.122**	30.05	0.042**	4.22	0.107**	23.66	0.047**	4.62	0.181**	13.10
	SY×SM	15	0.006**	63.41	0.051**	37.87	0.205**	62.15	0.067**	44.92	0.221**	65.35	0.243**	52.70
	Residuals	48	<0.001	7.57	0.010	22.45	0.005	5.25	0.009	19.89	0.006	5.28	0.015	10.12
Root	SY	3	0.007**	33.70	0.112**	24.65	0.294**	63.05	0.158**	28.08	0.272**	58.42	0.479**	61.34
	SM	5	0.002**	18.42	0.027**	9.98	0.034**	12.10	0.028**	8.23	0.036**	12.72	0.012**	2.49
	SY×SM	15	0.001**	28.06	0.055**	60.21	0.022**	23.95	0.065**	58.08	0.025**	27.12	0.050**	32.21
	Residuals	48	<0.001	19.82	0.001	5.16	<0.001	0.90	0.002	5.61	0.001	1.74	0.002	3.96

Type III sums of squares converted to percentages at each level. All data were log_10_-transformed before analysis; d.f.: degree of freedom, MS: mean squares, SS%: percentage of the sum of squares explained (%); ns indicates not significant (P > 0.05);

* indicates statistically significant at the 0.05 significance level (* P < 0.05 and ** P < 0.001).

**Fig 1:**
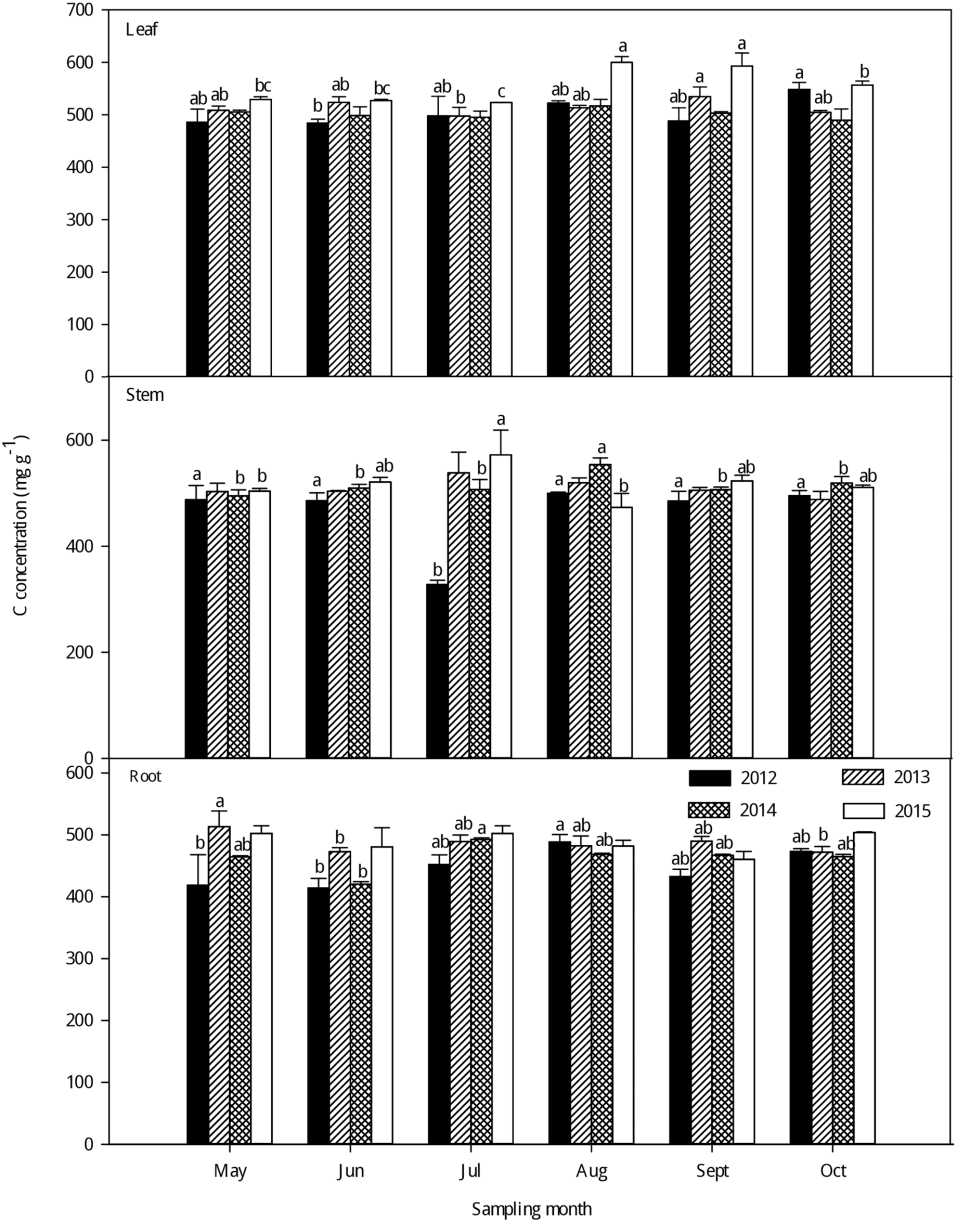
Seasonal variations in the C concentrations (mean ± SE) in different organs of larch stands from 2012–2015. Different lowercase letters represent significant differences among different growing seasons at P < 0.05.

**Fig 2:**
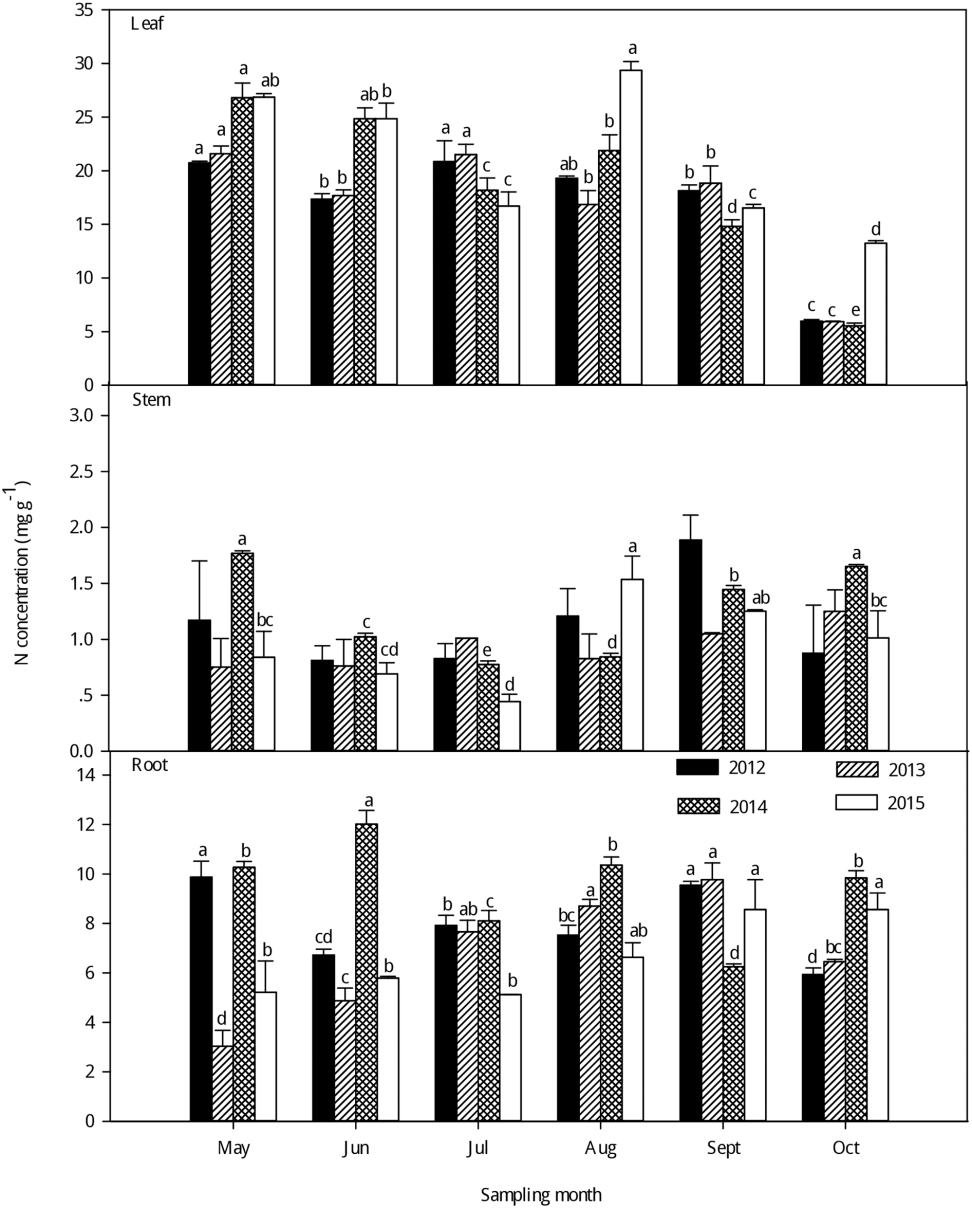
Seasonal variations in the N concentrations (mean ± SE) in different organs of larch stands from 2012–2015. Different lowercase letters represent significant differences among different growing seasons at P < 0.05.

**Fig 3:**
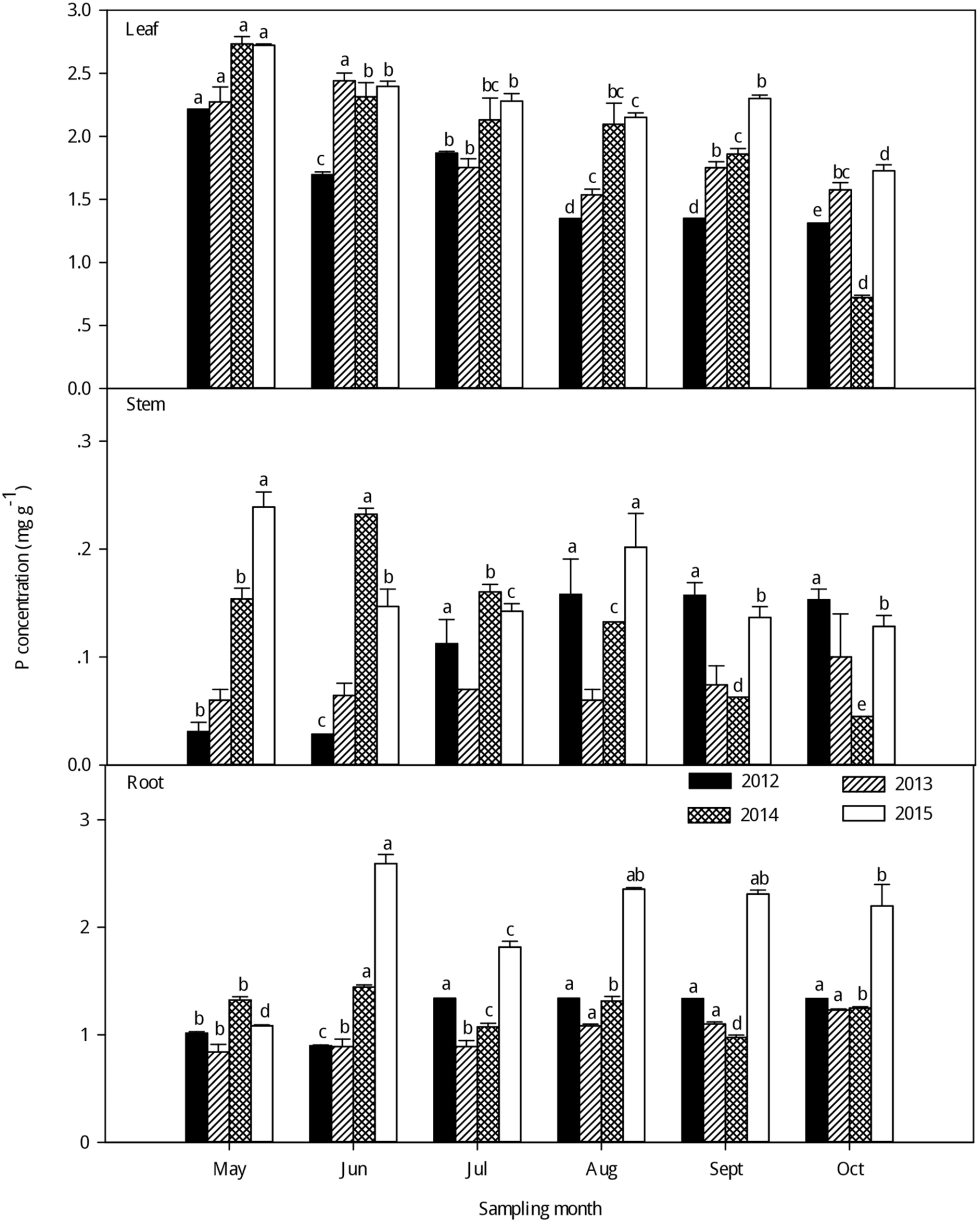
Seasonal variations in the P concentrations (mean ± SE) in different organs of larch stands from 2012–2015. Different lowercase letters represent significant differences among different growing seasons at P < 0.05.

As seen in Figs 1–3, large variations in C, N and P concentrations were observed among the different organs of the larch stands in the different growing seasons from 2012–2015. Generally, there was little change in the C concentrations of different plant organs among the different sampling months; the leaf had a higher C concentration than in the stem and root (P < 0.05) (Fig 1and S2 Table). With the growth of the larch stands in each sampling year, the C concentrations increased in the leaf and peaked in the fast-growing season. However, no clear seasonal tendencies in the stem and root C concentrations were found with growth. However, the N concentrations were significantly higher in the leaf than in the stem and root in each sampling year (P < 0.001) (Fig 2 and S2 Table), and the N concentrations in the leaf generally increased from the early growing season to the fast-growing season, the highest leaf N concentrations were observed in the fast-growing season and the lowest in the late-growing season. Additionally, a second peak in leaf N concentrations was observed in the early growing season. For the stem and root N concentrations, no clear seasonal tendencies were observed in the different growing seasons from 2012–2015, but there were large differences in the leaf, stem, and root P concentrations (Fig 3 and S2 Table). Generally, there was a higher P concentration in the leaf than in the stem and root (P < 0.001). The P concentrations in the leaf showed a gradual decreasing trend with growth, and the highest leaf P concentrations were observed in the early growing season while the lowest were in the late-growing season in each sampling year. No clear seasonal tendencies in the stem and root P concentrations were observed with the growing season from 2012–2015.

### Seasonal variations in the C:N:P stoichiometry of different organs of larch stands

Sampling month, sampling year (stand age), plant organ and their interactions significantly affected the C:N, C:P and N:P ratios in different organs of larch stands from 2012–2015. Based on GLM analysis, we found that plant organ was the largest contributor to the C:N and C:P ratios (89.53% and 90.09%, respectively), but the largest contributor to the N:P ratios was the interaction of sampling year × plant organ × sampling month (19.83%), followed by plant organ (18.93%), the sampling year × sampling month interaction (17.86%) and sampling year (15.17%) (Table 2). For the leaf C:N, C:P and N:P ratios, sampling month determined the greatest amount of the variation (82.95%, 63.54% and 55.90%, respectively), and the sampling year × sampling month interaction was the larger contributor to the leaf N:P (39.25%). The interaction of sampling year × sampling month was the largest contributor to the stem C:N, C:P and N:P ratios (44.92%, 65.35% and 52.70%, respectively). For the root C:P and N:P ratios, the largest contributor was sampling year (58.42% and 61.34%, respectively), while the largest contributor to the root C:N ratios was the interaction of sampling year × sampling month (58.08%) (Table 3).

**Fig 4:**
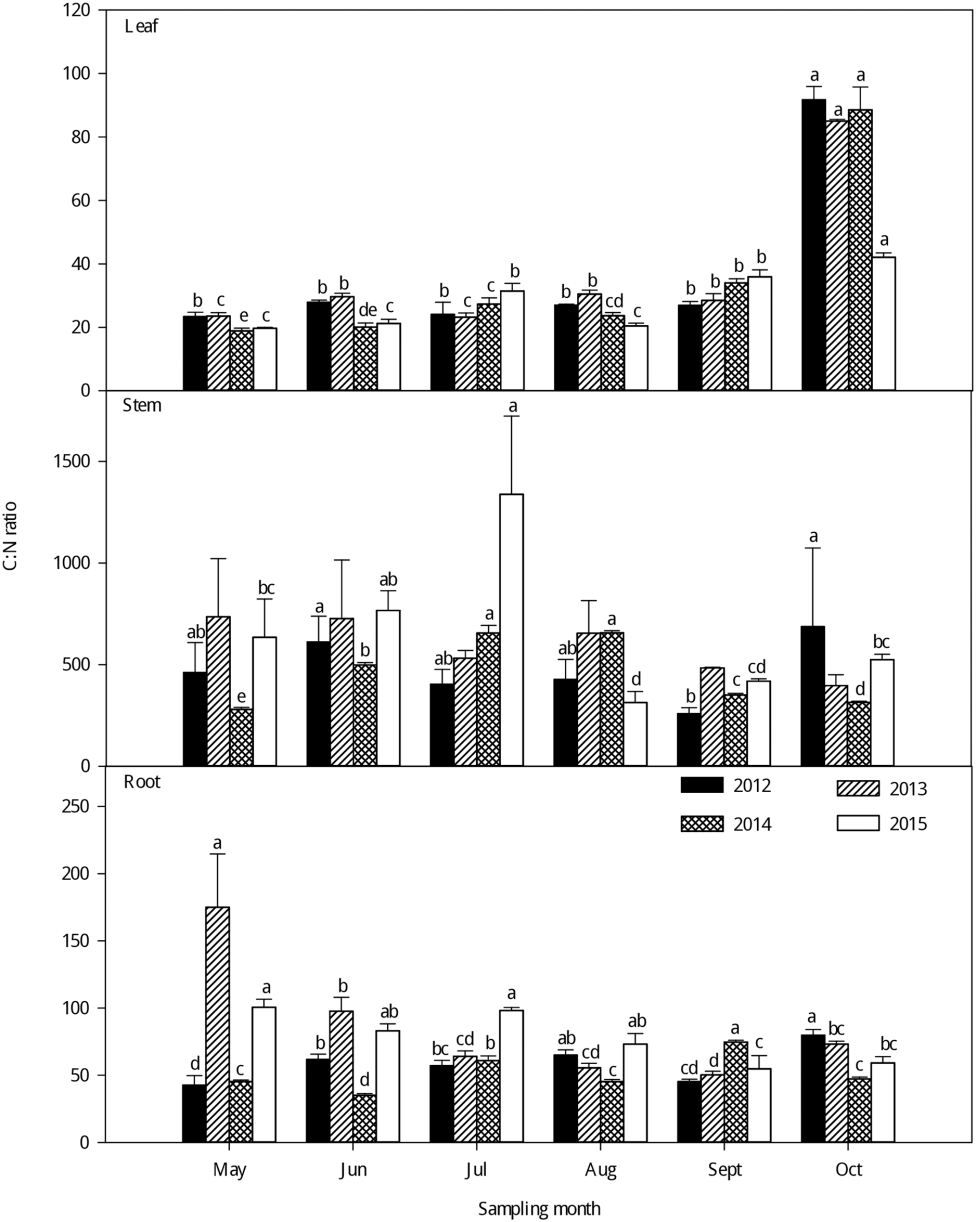
Seasonal variations in the C:N ratio (mean ± SE) in different organs of larch stands from 2012–2015. Different lowercase letters represent significant differences among different growing seasons at P < 0.05.

**Fig 5:**
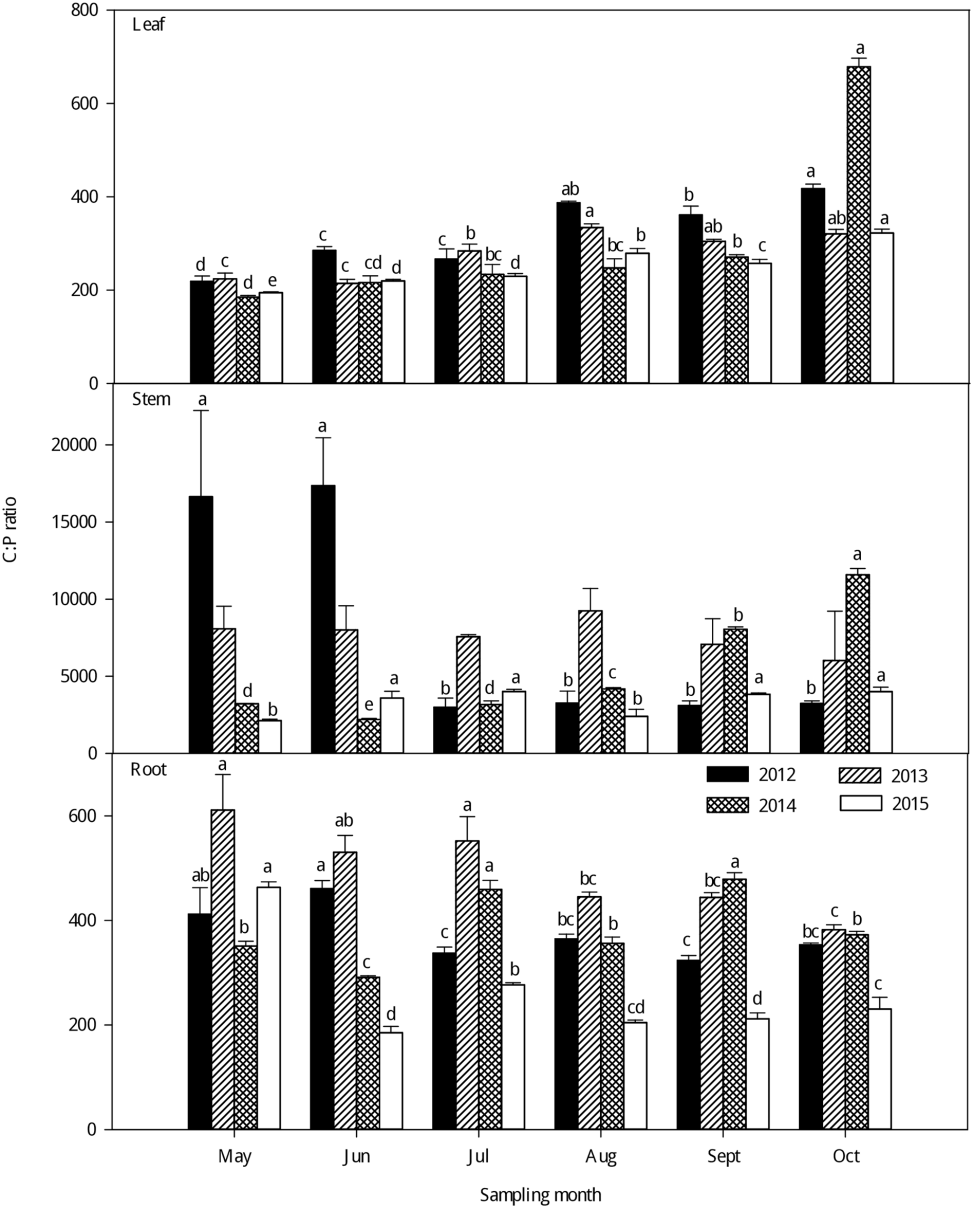
Seasonal variations in the C:P ratio (mean ± SE) in different organs of larch stands from 2012–2015. Different lowercase letters represent significant differences among different growing seasons at P < 0.05.

**Fig 6:**
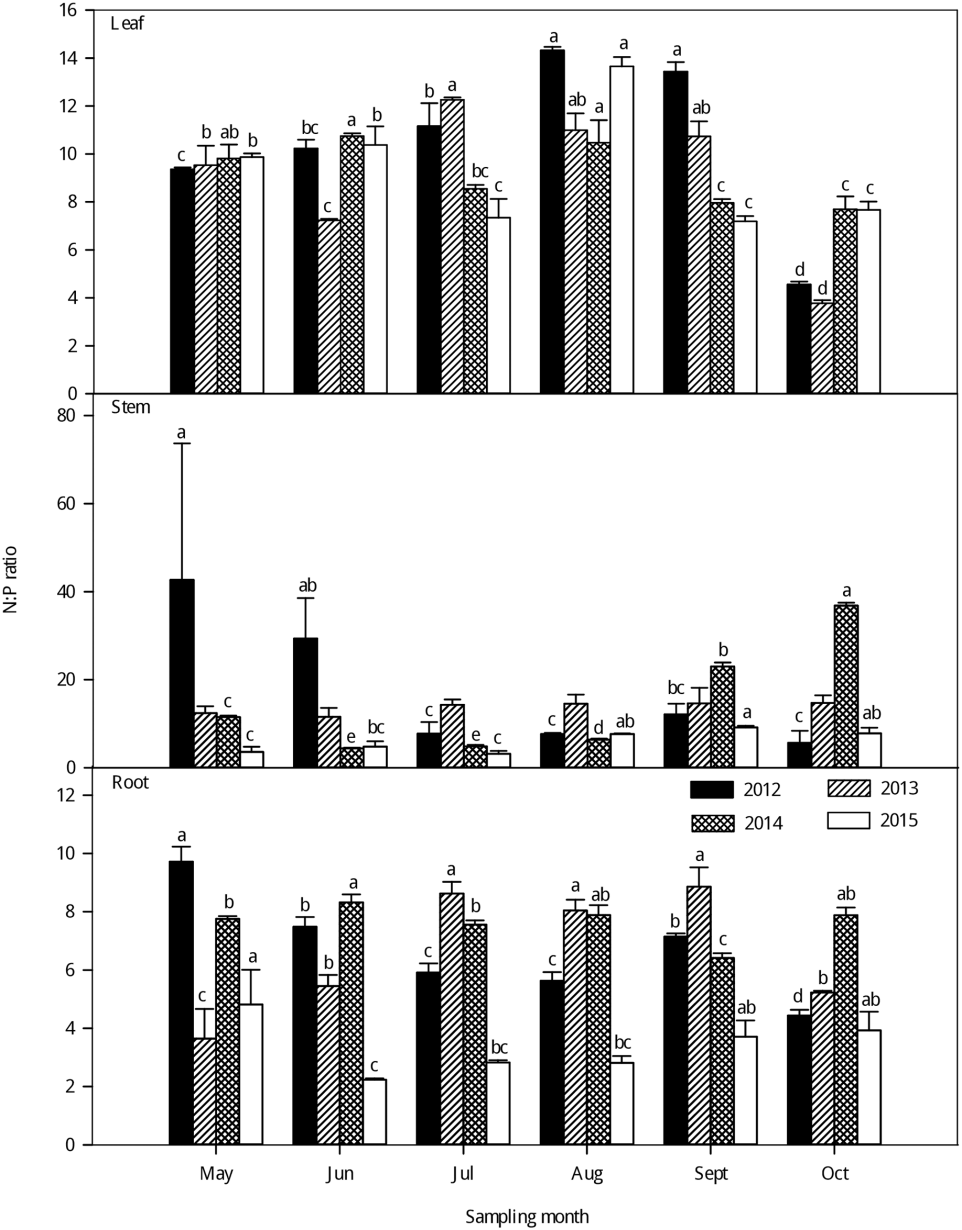
Seasonal variations in the N:P ratio (mean ± SE) in different organs of larch stands from 2012–2015. Different lowercase letters represent significant differences among different growing seasons at P < 0.05.

As seen in Figs 4–6, large variations were observed in the C:N, C:P and N:P ratios in the different organs of larch stands in different growing seasons from 2012–2015. Generally, the leaf C:N and C:P ratios significantly increased with growth from May to October from 2012–2015. The highest leaf C:N and C:P ratios were observed in the late-growing season and the lowest in the early growing season. However, no clear seasonal tendencies were observed in the C:N and C:P ratios of the stems and roots with the growth of larch stands from 2012–2015 (Figs 4 and 5). The C:N and C:P ratios were significantly higher in the stem than in the leaf and root and lowest in the leaf (P < 0.001) (S3 Table). There was little difference in the N:P of different plant organs among different seasons (Fig 6), but the leaf had higher N: P than the stem and root (P < 0.001) (S3 Table). Generally, the N:P ratios in different organs exhibited large seasonal fluctuations; the highest leaf N:P ratios occurred in the fast-growing season and the lowest in the late-growing season. For the stem and root N:P ratios, there were no clear seasonal tendencies with growing season.

### Relationships among C, N and P concentrations and the C:N, C:P and N:P ratios in different organs with stand age

In this study, C, N and P concentrations and their stoichiometric ratios were significantly affected by stand age. However, no monodirectional linear correlation of the different elements and their stoichiometric ratios was observed with increased stand age, and varying linear correlations were observed in different sampling months from 2012–2015 (Table 4). Generally, the leaf and stem C concentrations were positively correlated with increased stand age in all growing seasons (except for the leaf C in July and October). However, the root C concentrations was positively correlated with increased stand age in the early growing season (May and June) and the early of the fast-growing season (July), but there was a positive correlation in the mid-late of the fast growing season (August and September). For the N concentrations of different plant organs, varying linear correlations were observed in different sampling months. Interestingly, there was no significant correlation between the P concentrations of different plant organs in different growing seasons.

**Table 4:** Correlations of the C, N, P concentrations and the C:N, C:P, and N:P ratios in different organs with stand age in different sampling months from 2012–2015.

Element	Plant organ	Sampling month					
		May	Jun	Jul	Aug	Sept	Oct
C	**Leaf**	1.00***	1.00***	0.93^ns^	0.96*	1.00***	-1.00**
N		0.99*	0.99**	-0.99*	0.98*	0.87^ns^	0.48^ns^
P		0.92^ns^	0.77^ns^	0.87^ns^	0.96*	0.98*	0.41^ns^
C:N		-0.97*	-0.99*	1.00**	-0.99*	0.99**	-0.99**
C:P		-1.00***	0.98*	-1.00***	-1.00***	-0.95*	-1.00***
N:P		0.99*	0.99**	-0.99*	0.98*	0.87^ns^	0.48^ns^
C	**Stem**	1.00**	1.00***	1.00***	1.00**	1.00***	1.00**
N		0.25 ^ns^	0.12^ns^	0.76^ns^	0.39^ns^	0.68^ns^	0.30^ns^
P		0.98*	0.75^ns^	0.59^ns^	0.44^ns^	0.20^ns^	0.35^ns^
C:N		1.00***	1.00***	0.91^ns^	0.22^ns^	1.00***	-1.00***
C:P		0.95^ns^	0.89^ns^	1.00***	-1.00***	0.17^ns^	1.00***
N:P		0.25^ns^	0.12^ns^	0.76^ns^	0.39^ns^	0.68^ns^	0.30^ns^
C	**Root**	1.00**	1.00***	1.00**	-0.99*	-0.99*	0.75^ns^
N		-0.98*	0.66^ns^	0.94^ns^	0.96*	0.79^ns^	0.96*
P		0.44^ns^	0.91^ns^	0.52^ns^	0.75^ns^	0.71^ns^	0.75^ns^
C:N		1.00***	1.00**	0.99*	-1.00***	0.98*	-1.00***
C:P		1.00***	-1.00***	-1.00***	-1.00***	1.00***	-0.99**
N:P		-0.98*	0.66^ns^	0.94^ns^	0.96*	0.79^ns^	0.96*

An asterisk (*) indicates significant correlations at P < 0.05, double asterisks (**) indicates significant correlations at P < 0.01, and *** indicates significant correlations at P < 0.001. No significant at 5% level is shown by ns.

Generally, the linear correlations between the leaf C:N and C:P and the stand age in the fast-growing season and the late-growing season were similar to those of the root. In the early growing season (May and June), there were positive correlations between the stem and root C:N and stand age, while there were negative correlations in the leaf. For the C:N of different plant organs, there was a negative correlation with increased stand age in the late-growing season (October), but varying linear correlations were observed in the C:N of different plant organs in the fast-growing season (July, August and September). For the C:P ratio, negative correlations with increased stand age were observed in the leaf and root from the fast-growing season to the late-growing season (except for the root C:P in September) and varying linear correlations were observed in the early growing season. For the stem C:P, varying linear correlations were observed with increased stand age during entire growing season. Interestingly, a varying linear correlations between the N:P of different organs and stand age was observed among different growing season.

## Discussion

### Patterns of C, N and P concentrations in the leaf, stem and root

Compared with other studies that collected samples in July or July-September, the results of this study demonstrated that the leaf C concentrations of larch stands were higher than those of the arid saline biomes of China and the global terrestrial average [14, 28, 29] (Table 5). Furthermore, the leaf N concentrations were lower than those of the arid saline biomes of China [28] and the desert regions of north China [30] (Table 5). Generally, the leaf P concentrations were higher than those of the arid saline biomes of China, the Chinese flora and the global average [14, 19, 28, 29] (Table 5). Additionally, the mean root C concentrations (samples collected during July-September) were slightly lower than that of the global average [31] (Table 6). The root N concentrations were significantly lower than those of the global average and the grasslands of Inner Mongolia, China, while the root P concentrations were higher than those of the global average and the grasslands of Inner Mongolia, China [31, 32] (Table 6). The differences in the C, N and P concentrations among different organs are probably due to the different ecosystem types, sampling times, soil nutrients and different climatic factors among different studies. Many studies have reported organ-specific differences (among different plant organs) and site-specific differences (temperature, precipitation, etc.), that account for much of the variability in plant nutrient concentrations [33, 34]. In conclusion, the larch plantations in the Qinling Mountains had higher C:N and C:P ratios but a lower N:P ratio than those found in previous studies.

**Table 5:** Statistics of the leaf C, N, P concentrations (mg g^-1^) and C:N, C:P, and N:P ratios (mass ratio) (mean ± SD) in this study and previous studies.

Data source	Sampling month	Sampling year	C (mg g^-1^)	N (mg g^-1^)	P (mg g^-1^)	C:N	C:P	N:P
This study	July	2012	498.07±37.17	20.85±1.95	1.87±0.01	24.13±3.80	266.88±11.56	11.16±0.96
		2013	497.76±16.25	21.50±0.97	1.75±0.07	23.18±1.37	284.22±14.56	12.26±0.10
		2014	495.54±11.45	18.49±1.15	2.13±0.17	27.33±1.98	233.82±15.22	8.55±0.16
		2015	523.41±0.27	16.70±1.32	2.28±0.06	31.46±2.39	229.73±6.08	7.34±0.78
	Jul–Sept	2012	502.87±17.54	19.43±1.36	1.52±0.30	26.04±1.66	338.64±63.47	12.97±1.00
		2013	515.26±18.51	19.07±2.33	1.68±0.13	27.38±3.77	307.82±25.22	11.33±0.82
		2014	505.16±10.79	18.29±3.53	2.03±0.15	28.33±5.23	250.65±18.5	8.99±1.31
		2015	572.03±42.26	20.87±7.36	2.24±0.08	29.26±7.96	255.46±24.70	9.39±3.69
Wang (2015)	Jul		396.7±45.4	28.1±9.4	1.85±0.5	15.7±5.6	229.4±63.7	15.4±3.7
Li (2010)	Jul–Sept		-	24.5±8.1	1.74±0.9	-	-	15.77±7.5
Han (2005)	Jul–Sept		-	20.2±8.4	1.46±1.0	-	-	16.3±9.3
Reich (2004) and Elser (2000)	Jul–Sept		461.3±72.2	20.1±8.7	1.77±1.1	23.8±17.3	300.9±236.8	13.8±9.5

**Table 6:** Statistics of the root C, N, P concentrations (mg g^-1^) and C:N, C:P, and N:P ratios (mass ratio) in this study and previous studies.

Data source	Sampling month	Sampling year	C (mg g^-1^)	N (mg g^-1^)	P (mg g^-1^)	C:N	C:P	N:P
This study	Jul–Aug	2012	470.35±25.76	7.72±0.27	1.34	61.13±5.50	351.40±19.31	5.77±0.20
		2013	485.79±4.84	8.18±0.74	0.99±0.14	59.75±6.03	498.75±75.57	8.33±0.41
		2014	480.33±17.14	9.23±1.59	1.19±0.17	53.06±11.06	408.02±72.83	7.72±0.23
		2015	492.04±14.53	5.87±1.07	2.08±0.38	85.64±17.69	240.71±51.09	2.82±0.01
	Jul–Sept	2012	457.77±28.40	8.33±1.07	1.34	55.87±9.91	342.18±21.01	6.23±0.81
		2013	487.08±4.08	8.71±1.05	1.03±0.12	56.58±6.95	480.50±62.09	8.51±0.42
		2014	475.84±14.40	8.24±2.05	1.12±0.17	60.26±14.72	431.63±65.76	7.28±0.77
		2015	481.48±20.98	6.77±1.72	2.16±0.30	75.34±21.79	226.94±43.30	3.12±0.52
Jackson (1997)	Jul–Sept		488.0±9.5	11.7±0.73	1.1±0.17	41.71	443.64	10.64
Zhou (2010)	Jul–Aug		-	10.90	0.7	-	-	15.57

It is well known that C, N and P are the major nutritional elements required for plant growth. A complete description of the allocation of nutrients in different plant tissues is critical for explaining plant functional diversity [35]. The C, N and P concentrations of larch stands observed in this study were significantly co-affected by organ type, growing season and stand age. Furthermore, changes in the nutritional elements in different plant organs with ontogenetic development has been documented in many species. Additionally, seasonal variations in nutritional elements represent a dynamic growth response and may be related to the migration nutrients caused by changes in the balance between the element uptake and utilization efficiency, which may lead to different C, N and P concentrations in different plant organs [2, 3, 33]. In this study, unsynchronized variations in the C, N and P concentrations of different organs were observed in larch stands in the different growing seasons, but the C and N concentrations in the leaf during the fast-growing season were higher than those during the early or late-growing seasons. Faster growth may promote greater C and N accumulation via enhanced photosynthesis [36]. However, the highest leaf P concentrations occurred in the early growing season, and the lowest occurred in the late-growing season, which may have been related to the dilution of P concentrations with growth.

In addition, the N and P concentrations in the leaf were significantly higher than those in the stem and root, indicating that the allocation of nutritional elements to the leaf was prioritized compared with the stem and root. In plants, the leaf is the most important photosynthetic organ, and it requires high nutrient concentrations to improve photosynthetic and metabolic capacity, which may represent a response to ontogenetic development [37]. Generally, the transfer of nutritional elements from one plant organ to another affects the element concentrations in different organs. The transfer of N and P from the leaves to other specific plant tissues before leaf abscission is required for plant survival [38–40], and this process corresponded to the lowest leaf N and P concentrations that were observed in the late-growing season in this study. Generally, the leaf P concentrations were highest in the early growing season, which was related to the need for P-rich ribosomal RNA to initiate plant growth, whereas the leaf N concentrations peaked in the early growing season and the fast-growing season, which was related to the high demand for abundant N-rich proteins to maintain the rapid growth rate of plant [41, 42]. Overall, the results of this study indicated that the leaf N and P concentrations were more easily influenced by growing season, plant organ and stand age than the C concentration, which is consistent with the results of other studies [13–15].

Previous studies have indicated that the nutritional elements in plants may be affected by a dilution effect caused by increased plant size and biomass [16, 43, 44], and the concentrations of nutritional elements in the leaf and stem decrease with ontogenetic development [2, 3, 7, 45]. In this study, we found that the dilution of C, N and P varied in the different plant organs of larch stand, and that of P was greater than C and N, especially in the leaf. However, the C and N concentrations in different organs did not show a dilution effect. For the leaf C concentrations, slight increases were observed with growth, and this finding is inconsistent with the findings of Elser et al. (2010) [43]. Generally, the assimilative capacity of C is associated with growth [46], and in this study, the maximum C concentrations of different organs occurred in the fast-growing season, which indicated that the larch stands had the highest plant C assimilative capacity and the highest carbohydrate transport efficiency in the fast-growing season. The variations in the C, N and P concentrations in the leaf, stem and root were possibly due to the trade-off between the uptake and storage efficiency [33]. Seasonal patterns of variation in the C, N and P concentrations in the leaf, stem and root were linked to the remobilization and re-translocation of C, N and P from the root to the aboveground parts and from the stem to the leaf [47]. The results indicated that changes in the profiles of different nutrient elements in the different tissues of such perennials may be related to the nutrient use efficiency in different growing seasons.

### Patterns of the C:N:P stoichiometry in the leaf, stem and root

Compared with other studies that collected samples in the same sampling months, the results demonstrate that the leaf C:N and C:P were higher than those of the arid saline biomes of China and the global average for terrestrial plants [14, 28, 29] (Table 5). The leaf N:P ratios were significantly lower than those of the arid saline biomes of China [28], the desert regions of north China [30], the Chinese flora [19] and the global average [14, 29] (Table 5). Furthermore, the root C:N ratios were significantly lower than the global average [31] (Table 6), and the root N:P ratios were significantly lower than those of the global average [31] and the grasslands of Inner Mongolia, China [32] (Table 6). Generally, the root C:P ratios were lower than the global average [31], except in 2013 (Table 6). Many studies have shown that C:N:P stoichiometry can be affected by ecosystem type, abiotic factors (such as temperature, elevation, precipitation, and drought) [14, 35], and biological factors (such as species compositions, life form, and genotype) [43, 48]. In conclusion, the larch plantations in the Qinling Mountains had higher C:N but lower C:P and N:P ratios than those found in previous studies.

In this study, the C:N, C:P and N:P ratios were significantly co-affected by the organ type, stand age and growing season, and these factors may cause variations in the stoichiometry. In this study, significant differences were observed in the C:N, C:P and N:P ratios among different organs of larch stands, which was explained by differences in the N and P allocation patterns and the N- and P-use efficiencies during different growth stages [49, 50]. Across all plant organs, the C:N and C:P ratios in the stem were larger than those in the leaf and stem, indicating that the transmission organ (stem) possessed higher C:N, C:P and N:P ratios than the absorption organ (root) and the metabolic organ (leaf). However, large fluctuations in the N:P ratio were observed among different organs, and this may be explained by the N and P activity in plants (especially for plants within the fast-growth stage), both of which are not only readily affected by growing season, plant age or their interactions but also are influenced by other environmental factors, such as the size of plant organ [16].

C:N stoichiometry is used to indicate the plant N-use efficiency and response to C fixation and N assimilation, and it is positively correlated with plant N-use efficiency but negatively correlated with plant growth rate [51]. During the different growing seasons of larch stands in this study, different C and N statuses and different C-fixation and N-assimilation rates were observed in different organs. The leaf C:N increased with the growth of larch stands and peaked in the late growing season, which may be explained by increased leaf C accumulation but decreased N accumulation in the late-growing season. However, for the stem and root C:N ratios, no clear seasonal variations were observed. The C:N ratios were significantly higher in the stem than in the leaf and root in this study. Generally, the lowest C:N ratios were observed in the leaf, and the highest were in the stem, which may be related to the transfer of carbohydrates from photosynthetic to structural organs [51]. Previous studies have indicated that the increase in the proportion of stem biomass with plant growth may lead to increased C:N ratios in the structural organs of plants, resulting in a higher C:N ratio in the stem [51, 52], which is consistent with our results in this study.

C:P stoichiometry is used to indicate the P-use efficiency and the balance between C fixation and P assimilation in plants, and it is positively correlated with the plant P-use efficiency but negatively correlated with plant growth rate [51]. In forest ecosystems, an increase in woody biomass and lignification may lead to seasonal variations in C:P ratio at different growth stages and among different plant organs [12]. In this study, the leaf C:P ratios increased with the growing season and peaked in the late-growing season, and this may be explained by the growth, leading to the increased leaf C accumulation and the leaf P dilution. An opposite trend existed in the root in this study, and the root C:P ratios decreased with the growth of larch stands. However, no clear seasonal trends in the variation of the stem C:P ratios were observed throughout 2012–2015. In conclusion, the leaf C:N and C:P ratios peaked in the late-growing season, indicating the highest N- and P-use efficiency in the leaf but the lowest growth rate. Variation in the leaf C:N and C:P ratios with growth represents a dynamic response to the N- and P-use efficiency of plants at different growth stages and corresponds to patterns of plant ontogeny. Thus, the leaf C:N and C:P ratios increased in late-growing season, reflecting the accumulation of compounds with higher C-to-nutrient ratios.

The growth rate hypothesis (GRH) suggests that the N:P ratio is negatively correlated with plant growth rate [43]. In this study, the N:P ratios in different organs were flexible in different growing seasons. Generally, the leaf N:P ratios increased with the growth of larch stands throughout their life cycle, and the highest leaf N:P ratios occurred in the fast-growing season, indicating a low growth rate for larch stands in this growth stage. This finding was consistent with the results of other studies; Agren (2008) indicated that leaf N:P ratios decreased with plant growth in terrestrial ecosystems [10]. However, Orgeas et al. (2003) found that leaf N:P ratios initially increased, then decreased, and finally tended to stabilize from the early growing season to the fast-growing season [53]. Thus, further studies are required to clarify these differences. Across the entire growing season, no clear seasonal trends were observed in the variation in the stem and root N:P ratios in our study, which may be related to the stem acting as the transmission tissue between the leaf and root. Therefore, the N:P ratios were affected by the N- and P- uptake efficiency in the root, the N- and P- utilization efficiency in the leaf and the N and P transfer efficiency in the stem.

The leaf N:P ratio can be used to determine potential N or P limitations in plants [46, 54, 55]. A ratio <14 indicates N limitation, and a ratio >16 indicates P limitation. In this study, the leaf N:P ratios of larch stands were generally lower than 14 during the entire growing season from 2012–2015. Considering the soil nutrient background of our study region (Table 1), the growth of larch plantations was much more limited by N than by P. However, in some months during 2012–2015, the leaf N:P ratios were between 14 and 16, indicating equal limitation of larch growth by N and P [56]. Thus, the results indicated that the type and intensity of nutrient limitation in plants may change with the growing season. Additionally, in terrestrial ecosystems, N and P concentrations in different plant organs are affected by the N and P concentrations in the soil [14]. Plant N and P are derived from the soil pool, and the C, N and P concentrations in soil mainly depend on the release of C, N and P through litter decomposition by microbes. Therefore, changes in the soil N and P concentrations could potentially affect leaf C:N:P stoichiometric ratios [3]. However, the litter is difficult to decompose by microbes in soil when with a C:N ratio >25 [57]. In this study, the C:N ratios of different plant organs were significantly greater than 25 in the late-growing season, which blocked the C, N and P cycling in the soil in the study region. This probably led to the growth of larch plantation being more severely limited by the N. Therefore, the optimal application of N fertilizer is practical and will be helpful for sustaining the productivity of larch plantations in the study region. Additionally, we found a similar trend in N:P variation in the leaf and root, indicating that the root N:P ratio may also be used as an indicator to infer potential N- or P- limitation in plants.

### Correlation study

Understanding how increases in stand age affects C, N and P concentrations and stoichiometry represents one of the most important aspects of forest management [58]. In this study, the C, N and P concentrations and their stoichiometric ratios in different organs of larch stands were significantly affected by stand age, and varying linear correlations with stand age were observed in the different growing seasons from 2012–2015. In this study, the leaf and stem C concentrations significantly increased with increased stand age, which was explained by the growth being most active in young plant organs. Thus, C fixation is stronger in young plant organs than in old organs. However, no clear correlation was observed among different growing seasons between the N and P concentrations of different plant organs and stand age in this study, which was explained by the N and P concentrations in plant tissue being more easily affected by various factors. Markovic et al. (2009) found that the N concentrations decreased and the P concentrations increased in part or all tissues of lucerne with the growth [59], which is consistent with our results. Chen et al. (2004) found that leaf P concentrations of *Pinus sylvestris* var. decreased with increased stand age and the leaf N concentrations of a *Pinus* spp. plantation did not show a consistent change with stand age [60]. However, in our study, no significant tendency was observed in the leaf N and P concentrations with increased stand age in different sampling months. This may be explained by the N and P activity in plants not only being easily affected by sampling time, plant age and their interactions but also influenced by soil nutrients [16]. In conclusion, we found that the changes in different nutritional elements in perennials with increased stand age may change due to differences in the utilization efficiency of nutritional elements, and although the effects of stand age on the concentrations of different elements are considerable, they are not completely understood. Our study revealed that the main nutritional elements (C, N, and P) in larch plantation may change in different growing seasons and with increased stand age, although a more complete description of the changes in nutritional elements is required.

Previous studies have indicated that the increased woody biomass and increased lignification of plants in forest ecosystems may lead to an increase in the C:N and C:P ratios of different tissues with stand age [12, 52]. However, no monodirectional linear correlations were observed between the C:N, C:P and N:P ratios in different tissues and stand age during different growing seasons from 2012–2015 in our study. Overall, the leaf and root C:N ratios increased with increased stand age in the fast-growing season (except for August), and this was consistent with the report by Yang and Luo (2011) [12], who found that leaf C:N ratios increased with increased stand age, and that the tissues C:P of forest plants also increased with stand age. However, in this study, we found that the leaf and root C:P ratios decreased significantly with stand age over the entire life cycle of larch stands. Furthermore, multiple correlations were observed between the stem C:N and C:P ratios and stand age in different growing seasons.

Wang et al. (2014) indicated that the leaf N:P ratios of lucerne decreased and then increased with stand age [13]. However in this study, we found that the leaf N:P ratios increased with increased stand age in the early growing season, decreased in the early stage of the fast-growing season and then increased in the mid-stage of the fast-growing season. In terms of the stem and root N:P ratios, multiple correlations were observed with stand age in different growing seasons partly because N and P are very active in plant organs and thus easily influenced by stand age [13] or abiotic factors[14, 15]. Therefore, the N:P ratios of different organs were very flexible and the trends in the variation differed with increased stand age. The N:P ratio has proved to be a new, efficient but relatively easy way to assess plant N or P limitation [55, 61]. However for the same species with different stand ages, the N:P thresholds for judging N or P growth limitation may change with increased stand age.

## Conclusions

In contrast to natural forests, nutrient cycling within plantation forests can depend on artificial management, and the seasonal variations in C, N and P concentrations and their stoichiometric ratios in different growth stages can reflect the physiological requirements of the plants. However, short-term data could limit the ability to predict nutritional limitations in forest ecosystems, so we attempted to elucidate the tendency of the variation in C, N and P concentrations and their stoichiometries via long-term field experiments in a larch plantation. The results of our study suggest that the variation in the C, N and P concentrations and the C:N:P stoichiometric ratios in different plant organs may be caused by the variation in nutrient allocation patterns or physiological demands in different growing seasons, and the evidence indicated that nutrient limitation in forest ecosystems is a complex phenomenon. Thus, the use of short-term data to judge N or P limitation to plant growth is limited.

Our study indicated that the C,N and P concentrations and the C:N and C:P ratios in different plant organs were strongly affected by sampling time and plant age (stand age), and should be considered when compiling datasets across large spatial scales. To address the variation caused by differences in plant development and plant age, we suggest that additional information about these factors should become part of ecological plant stoichiometry. The C, N and P concentrations and the C:N, C:P and N:P ratios in different plant organs are significantly influenced by plant age (stand age), which may be used as a new methodology to understand plant growth and the formation of plant communities.

Our study indicated that the leaf N:P ratios varied with growing season and stand age. During certain periods of the growing season, the tissue N:P ratio significantly increased with stand age, and the leaf N:P ratios may continue to increase with increasing stand age and to values higher than 14. This topic is being investigated in a new ongoing project, and further studies are under consideration. Collectively, our results should be applicable to sustainable of larch plantation management and inform the application of optimal N fertilization to stands of all ages in the early growing season in our study region. Further investigations of the relationship between the stoichiometric ratios in plants and the growing season or stand age should be conducted to provide insights into the conservation and management of plantation forests.

## Supporting information

S1 Table Data are used in this study. (XLSX)

S2 Table Mean ± SE of the C, N and P concentrations in different organs of *L*. *principis-rupprechtii* Mayr. in different growing seasons from 2012–2015. (DOCX)

S3 Table Mean ± SE of the C:N, C:P and N:P ratios in different tissues of *L*. *principis-rupprechtii* Mayr. in different growing seasons from 2012–2015. (DOCX)

S1 Fig Study site. (TIF)

S1 File Relationship between the C, N and P concentration (S2 Fig, S3 Fig and S4 Fig, respectively) and C:N:P stoichiometry (S5 Fig, S6 Fig and S7 Fig, respectively) of different organs of *L*. *principis-rupprechtii* Mayr. and the stand age. Line indicate that the liner regressions were significant at P <0.05.
(ZIP)

S2 File Analysis of variance for the C, N and P concentrations (S4 Table) and the C:N, C:P and N:P ratios (S5 Table) of different organs of *L*. *principis-rupprechtii* Mayr. at various growing stages from 2012–2015. (ZIP)
