# Supplementary material for: Seasonal variations in carbon, nitrogen and phosphorus concentrations and C:N:P stoichiometry in different organs of a Larix principis-rupprechtii Mayr. plantation in the Qinling Mountains, China
Source: PLoS One. 2017 Sep 22;12(9):e0185163. doi: 10.1371/journal.pone.0185163 (PMC5609765; doi:10.1371/journal.pone.0185163)
Supplement: S2 Table — (DOCX) [file pone.0185163.s002.docx]

**S2 Table. Mean ± SE of the C, N and P concentrations of different organs of *L*. *principis-rupprechtii* Mayr. in different growing seasons from 2012-2015**

| **Element** | **Year** | **Plant organ** | **C concentration in the leaf, stem and root in different growing season (mg g^-1^)** | | | | | |
| --- | --- | --- | --- | --- | --- | --- | --- | --- |
|  |  |  | **May** | **June** | **July** | **August** | **September** | **October** |
| **C** | **2012** | Leaf | 485.76±25.00 ab | 484.07±7.40 b A | 498.07±37.17 ab A | 522.31±4.52 ab A | 488.22±25.00 ab A | 548.44±13.20 a A |
|  |  | Stem | 487.69±26.90 b | 485.92±14.86 b A | 327.98±8.24 a B | 500.00±2.11 b A | 485.68±17.94 b A | 495.44±9.57 b B |
|  |  | Root | 418.56±49.28 b | 414.11±15.39 b B | 452.13±15.54 ab A | 488.56±11.88 a B | 432.61±11.78 ab B | 473.11±4.45 ab B |
|  | **2013** | Leaf | 508.67±7.88 ab | 523.67±10.66 ab A | 497.76±16.25 b | 513.38±4.43 ab A | 534.64±18.51 a A | 504.76±3.25 ab A |
|  |  | Stem | 503.08±15.87 | 503.81±1.16 B | 538.46±39.05 | 519.66±9.41 A | 505.69±5.33 AB | 488.35±15.17 AB |
|  |  | Root | 513.13±25.37 a | 472.66±6.47 b C | 489.22±10.46 ab | 482.37±15.67 ab B | 489.64±7.76 ab B | 472.06±8.96 b B |
|  | **2014** | Leaf | 505.41±3.56 A | 498.52±16.60 A | 495.54±11.45 | 516.83±12.76 B | 503.12±2.78 A | 489.62±21.58 AB |
|  |  | Stem | 495.00±11.31 b A | 509.67±7.17 b A | 507.17±18.77 b | 553.96±12.65 a A | 506.70±4.93 b A | 519.14±12.56 b A |
|  |  | Root | 464.47±1.74 ab B | 420.26±4.20 b B | 492.45±2.60 a | 468.21±1.89 ab C | 466.86±1.88 ab B | 464.80±3.82 ab B |
|  | **2015** | Leaf | 529.18±5.28 bc A | 526.86±2.68 bc | 523.41±0.27 c AB | 599.95±10.92 a A | 592.74±24.98 a A | 556.67±7.51 b A |
|  |  | Stem | 504.11±5.01 b B | 521.18±8.95 ab | 572.32±46.58 a A | 473.45±26.29 b B | 523.26±10.80 ab B | 511.10±4.24 ab B |
|  |  | Root | 502.32±12.22 B | 480.32±31.14 | 502.32±12.22 B | 481.77±9.36 B | 460.36±12.75 C | 503.58±1.30 B |
| **N** | **2012** | Leaf | 20.74±0.16 a A | 17.35±0.51 b A | 20.85±1.95 a A | 19.30±0.21 ab A | 18.13±0.55 b A | 5.98±0.15 c A |
|  |  | Stem | 1.17±0.53 C | 0.81±0.13 C | 0.83±0.13 C | 1.21±0.25 C | 1.89±0.22 C | 0.88±0.43 B |
|  |  | Root | 9.87±0.65 a B | 6.72±0.24 cd B | 7.92±0.42 b B | 7.53±0.40 bc B | 9.55±0.16 a B | 5.93±0.27 d A |
|  | **2013** | Leaf | 21.59±0.72 a A | 17.68±0.53 b A | 21.50±0.97 a A | 16.86±0.57 b A | 18.84±1.61 b A | 5.94±0.04 c A |
|  |  | Stem | 0.75±0.26 C | 0.76±0.24 C | 1.01 C | 0.83±0.22 C | 1.05±0.01 C | 1.25±0.19 B |
|  |  | Root | 3.03±0.65 d B | 4.87±0.51 c B | 7.66±0.47 ab B | 8.70±0.27 a B | 9.77±0.68 a B | 6.46±0.09 bc A |
|  | **2014** | Leaf | 26.81±1.37 a A | 24.85±1.02 ab A | 18.49±1.15 c A | 21.88±1.48 b A | 14.82±0.61 d A | 5.54±0.25 e A |
|  |  | Stem | 1.77±0.02 a C | 1.02±0.03 c C | 0.77±0.03 e C | 0.84±0.03 d C | 1.45±0.04 b C | 1.65±0.02 a C |
|  |  | Root | 10.27±0.24 b B | 12.01±0.55 a B | 8.10±0.42 c B | 10.36±0.33 b B | 6.25±0.11 d B | 9.84±0.30 b B |
|  | **2015** | Leaf | 26.87±0.32 ab A | 24.85±1.46 b A | 16.70±1.32 c A | 29.37±0.81 a A | 16.53±0.34 c A | 13.23±0.25 d A |
|  |  | Stem | 0.84±0.23 bc C | 0.69±0.10 cd C | 0.44±0.08 d C | 1.53±0.21 a C | 1.25±0.01 ab C | 1.01±0.24 bc C |
|  |  | Root | 5.22±1.27 b B | 5.79±0.07 b B | 5.12±0.01 b B | 6.63±0.59 ab B | 8.56±1.21 a B | 8.56±0.67 a B |
| **P** | **2012** | Leaf | 2.22 a A | 1.70±0.02 c A | 1.87±0.01 b A | 1.35 d A | 1.35 d A | 1.31 e A |
|  |  | Stem | 0.03±0.01 b C | 0.03 b C | 0.11±0.02 a C | 0.16±0.03 a B | 0.16±0.01 a B | 0.15±0.01 a B |
|  |  | Root | 1.02±0.01 b B | 0.90±0.01 c B | 1.34 a B | 1.34 a A | 1.34 a A | 1.34 a A |
|  | **2013** | Leaf | 2.27±0.12 a A | 2.44±0.09 a A | 1.75±0.07 b A | 1.54±0.05 c A | 1.75±0.06 b A | 1.58±0.06 bc A |
|  |  | Stem | 0.06±0.01 C | 0.06±0.01 C | 0.07 C | 0.06±0.01 C | 0.07±0.02 C | 0.10±0.04 B |
|  |  | Root | 0.84±0.07 b B | 0.89±0.07 b B | 0.89±0.06 b B | 1.08±0.02 a B | 1.10±0.02 a B | 1.23±0.01 a A |
|  | **2014** | Leaf | 2.73±0.06 a A | 2.31±0.11 b A | 2.13±0.17 bc A | 2.10±0.17 bc A | 1.86±0.02 c A | 0.72±0.02 d B |
|  |  | Stem | 0.15±0.01 b C | 0.23±0.01 a C | 0.16±0.01 b C | 0.13 c C | 0.06 d C | 0.04 e C |
|  |  | Root | 1.32±0.03 b B | 1.44±0.02 a B | 1.07±0.03 c B | 1.31±0.04 b B | 0.98±0.02 d B | 1.25±0.01 b A |
|  | **2015** | Leaf | 2.72±0.01 a A | 2.40±0.04 b A | 2.28±0.06 b A | 2.15±0.04 c A | 2.30±0.03 b A | 1.73±0.05 d A |
|  |  | Stem | 0.24±0.01 a C | 0.15±0.02 b B | 0.14±0.01 b C | 0.20±0.03 a B | 0.14±0.01 b B | 0.13±0.01 b C |
|  |  | Root | 1.08±0.01 d B | 2.59±0.09 a A | 1.81±0.06 c B | 2.36±0.01 ab A | 2.31±0.04 ab A | 2.20±0.20 b B |

Values are the mean ± standard deviation of the mean (n=3). Lowercase letters represent significant differences among different months at P<0.05. Capital letters represent significant differences among different organs at P<0.05. The same letters indicate no significant difference at P<0.05.
