# Supplementary material for: Seasonal variations in carbon, nitrogen and phosphorus concentrations and C:N:P stoichiometry in different organs of a Larix principis-rupprechtii Mayr. plantation in the Qinling Mountains, China
Source: PLoS One. 2017 Sep 22;12(9):e0185163. doi: 10.1371/journal.pone.0185163 (PMC5609765; doi:10.1371/journal.pone.0185163)
Supplement: S3 Table — (DOCX) [file pone.0185163.s003.docx]

**S3 Table. Mean ± SE of the C:N, C:P and N:P ratios of different organs of *L*. *principis-rupprechtii* Mayr. in different growing seasons from 2012-2015**

| **Element** | **Year** | **Plant organ** | **C:N ratio in the leaf, stem and root in different growing season (mass)** | | | | | |
| --- | --- | --- | --- | --- | --- | --- | --- | --- |
|  |  |  | **May** | **June** | **July** | **August** | **September** | **October** |
| **C:N** | **2012** | Leaf | 23.43±1.35 b C | 27.91±0.65 b C | 24.13±3.80 b C | 27.06±0.25 b C | 26.93±1.21 b C | 91.74±4.21 a B |
|  |  | Stem | 461.48±147.82 ab A | 612.98±125.46 a A | 403.66±72.63 ab A | 427.35±98.82 ab A | 259.41±28.33 b A | 687.86±386.51 a A |
|  |  | Root | 42.64±7.02 d B | 61.71±3.99 b B | 57.24±3.86 bc B | 65.02±3.83 ab B | 45.34±1.78 cd B | 79.85±4.10 a B |
|  | **2013** | Leaf | 23.58±1.05 c C | 29.64±1.13 b C | 23.18±1.37 c C | 30.47±1.23 b C | 28.49±2.11 b C | 85.01±0.51 a B |
|  |  | Stem | 735.56±286.79 A | 726.88±288.62 A | 532.55±38.40 A | 655.42±160.12 A | 483.84±3.33 A | 396.41±53.94 A |
|  |  | Root | 174.93±39.73 a B | 97.73±10.24 b B | 64.01±4.12 cd B | 55.49±3.41 cd B | 50.25±2.69 d B | 73.15±2.23 bc B |
|  | **2014** | Leaf | 18.88±0.88 e C | 20.09±1.30 de C | 27.33±1.98 c C | 23.67±0.98 cd C | 33.99±1.30 b C | 88.53±7.22 a B |
|  |  | Stem | 279.96±9.23 e A | 499.24±11.47 b A | 656.30±37.83 a A | 657.03±10.67 a A | 350.69±7.84 c A | 314.29±5.49 d A |
|  |  | Root | 45.26±1.11 c B | 35.02±1.33 d B | 60.88±3.45 b B | 45.24±1.64 c B | 74.67±1.34 a B | 47.28±1.33 c C |
|  | **2015** | Leaf | 19.70±0.29 c C | 21.25±1.30 c C | 31.46±2.39 b C | 20.44±0.91 c C | 35.89±2.25 b C | 42.08±1.40 a B |
|  |  | Stem | 635.02±188.84 bc A | 766.89±97.38 ab A | 1338.90±384.31a A | 313.33±54.43 d A | 418.37±11.87 cd A | 524.89±127.06 bc A |
|  |  | Root | 100.51±26.09 a B | 83.02±5.22 ab B | 98.15±2.30 a B | 73.13±7.92 ab B | 54.73±9.87 c B | 59.11±4.71 bc B |
| **C:P** | **2012** | Leaf | 219.28±11.06 d C | 285.44±7.92 c C | 266.88±21.56 c B | 387.44±3.34 ab B | 361.60±18.45 b B | 417.92±9.44 a B |
|  |  | Stem | 16653.07±5580.86 a A | 17367.02±3101.91 a A | 2998.17±581.69 b A | 3268.49±768.05 b A | 3101.02±304.44 b A | 3243.49±163.21 b A |
|  |  | Root | 412.29±50.45 ab B | 461.00±15.41 a B | 337.74±11.36 c B | 365.06±8.85 bc B | 323.75±9.1 c B | 353.65±3.33 bc C |
|  | **2013** | Leaf | 224.26±12.27 c C | 214.48±8.87 c C | 284.22±14.56 b C | 334.41±7.62 a C | 304.83±4.16 ab C | 320.65±9.76 ab B |
|  |  | Stem | 8088.03±1461.46 A | 8006.60±1569.0 A | 7575.60±128.63 A | 9240.52±1457.41 A | 7072.58±1658.95 A | 6014.88±3208.00 A |
|  |  | Root | 611.11±68.15 a B | 530.36±32.38 ab B | 552.19±46.46 a B | 445.32±9.16 bc B | 443.99±9.3 bc B | 382.32±9.53 c B |
|  | **2014** | Leaf | 185.01±3.60 d C | 215.93±14.81 cd C | 233.82±21.22 bc C | 247.60±19.65 bc C | 270.53±5.50 b C | 678.75±18.39 a B |
|  |  | Stem | 3217.99±12.65 d A | 2194.90±68.25 e A | 3167.73±237.72 d A | 4174.65±90.04 c A | 8057.55±144.02 b A | 11584.06±403.33 a A |
|  |  | Root | 350.97±9.16 b B | 291.20±3.05 c B | 459.52±17.13 a B | 356.52±11.84 b B | 478.85±12.51 a B | 372.41±6.77 b B |
|  | **2015** | Leaf | 194.43±1.96 e C | 219.86±3.54 d B | 229.73±6.08 d C | 278.97±10.56 b B | 257.68±8.43 c B | 322.59±8.42 a B |
|  |  | Stem | 2114.19±103.52 b A | 3579.58±449.9 a A | 4010.82±134.08 a A | 2394.81±461.12 b A | 3829.84±85.78 a A | 3995.52±298.38 a A |
|  |  | Root | 463.28±10.54 a B | 185.37±11.89 d B | 276.84±4.13 b B | 204.59±5.09 cd C | 211.87±11.33 d C | 230.50±22.41 c C |
| **N:P** | **2012** | Leaf | 9.36±0.08 c B | 10.23±0.37 bc B | 11.16±0.96 b A | 14.32±0.15 a A | 13.43±0.4 a A | 4.56±0.12 d |
|  |  | Stem | 42.67±31.02 a A | 29.36±9.18 ab A | 7.72±2.62 c AB | 7.65±0.25 c B | 12.11±2.42 bc A | 5.67±2.7 c |
|  |  | Root | 9.72±0.52 a B | 7.48±0.33 b B | 5.91±0.31 c B | 5.63±0.3 c C | 7.14±0.11 b B | 4.44±0.2 d |
|  | **2013** | Leaf | 9.53±0.81 b A | 7.24±0.06 c A | 12.26±0.1 a B | 10.99±0.7 ab AB | 10.73±0.63 ab AB | 3.77±0.13 d B |
|  |  | Stem | 12.42±5.53 A | 11.54±2.03 B | 14.28±1.21 A | 14.53±3.07 A | 14.63±3.51 A | 14.68±5.74 A |
|  |  | Root | 3.64±1.02 c B | 5.45±0.38 b A | 8.62±0.4 a C | 8.04±0.37 a B | 8.86±0.66 a B | 5.23±0.06 b B |
|  | **2014** | Leaf | 9.81±0.58 ab B | 10.75±0.12 a A | 8.55±0.16 bc A | 10.47±0.94 a A | 7.96±0.16 c B | 7.69±0.54 c B |
|  |  | Stem | 11.50±0.33 c A | 4.40±0.15 e C | 4.83±0.35 e C | 6.36±0.23 d C | 22.99±0.92 b A | 36.85±0.68 a A |
|  |  | Root | 7.75±0.09 b C | 8.32±0.27 a B | 7.55±0.15 b B | 7.89±0.34 ab B | 6.41±0.16 c C | 7.88±0.26 ab B |
|  | **2015** | Leaf | 9.87±0.15 b A | 10.38±0.78 b A | 7.34±0.78 c A | 13.65±0.39 a A | 7.19±0.22 c B | 7.67±0.34 c A |
|  |  | Stem | 3.56±1.18 c B | 4.77±1.23 bc B | 3.13±0.69 c B | 7.63±0.15 ab B | 9.16±0.37 a A | 7.82±1.27 ab A |
|  |  | Root | 4.81±1.19 a B | 2.23±0.05 c C | 2.82±0.08 bc B | 2.81±0.23 bc C | 3.71±0.56 ab C | 3.93±0.64 ab B |

Values are the mean ± standard deviation of the mean (n=3). Lowercase letters represent significant differences among different months at P<0.05. Capital letters represent significant differences among different organs at P<0.05. The same letters indicate no significant difference at P<0.05.
