# Supplementary figures and images for: Seasonal variations in carbon, nitrogen and phosphorus concentrations and C:N:P stoichiometry in different organs of a Larix principis-rupprechtii Mayr. plantation in the Qinling Mountains, China
Source: PLoS One. 2017 Sep 22;12(9):e0185163. doi: 10.1371/journal.pone.0185163 (PMC5609765; doi:10.1371/journal.pone.0185163)

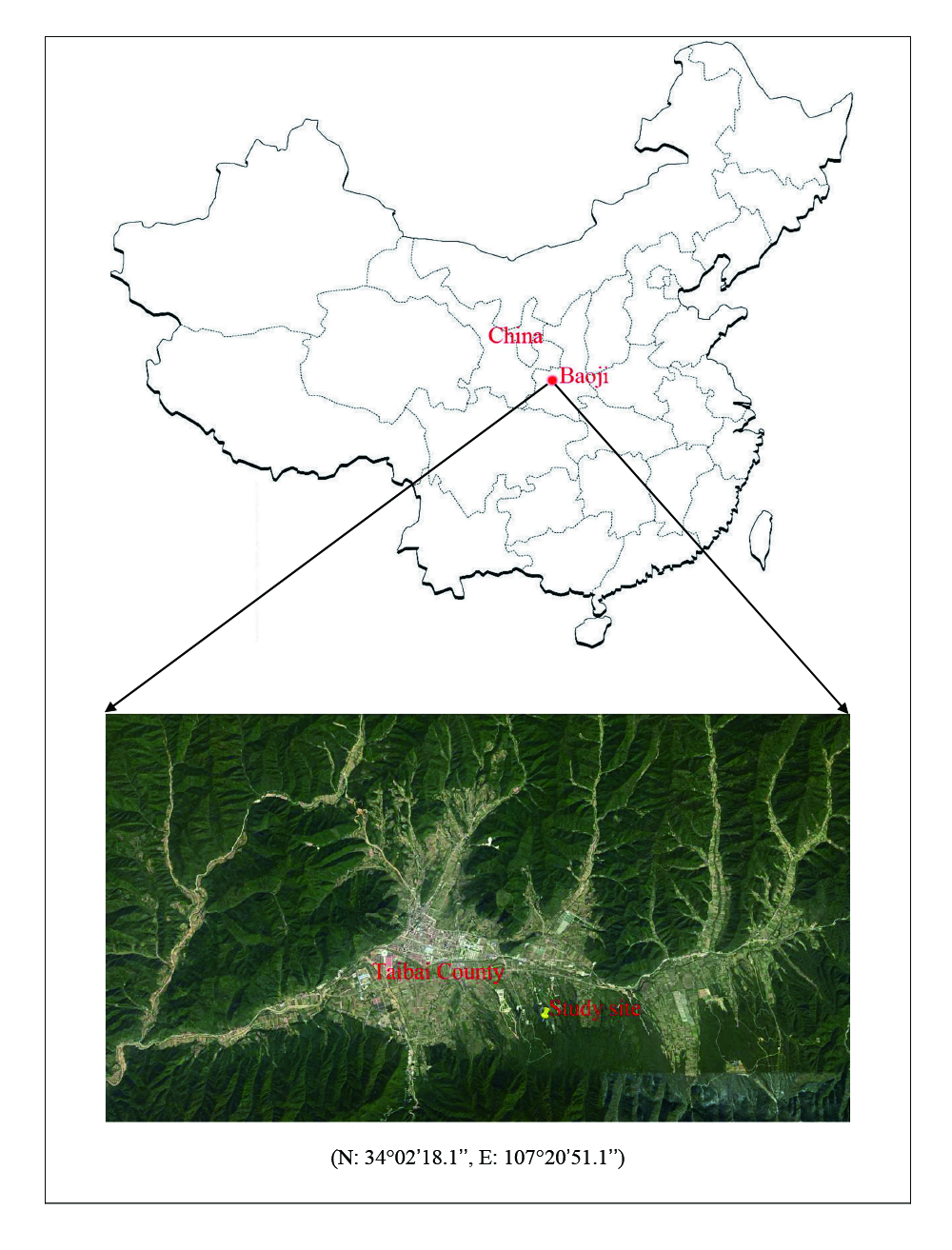

Supplement: S1 Fig — (TIF) [file pone.0185163.s004.tif]

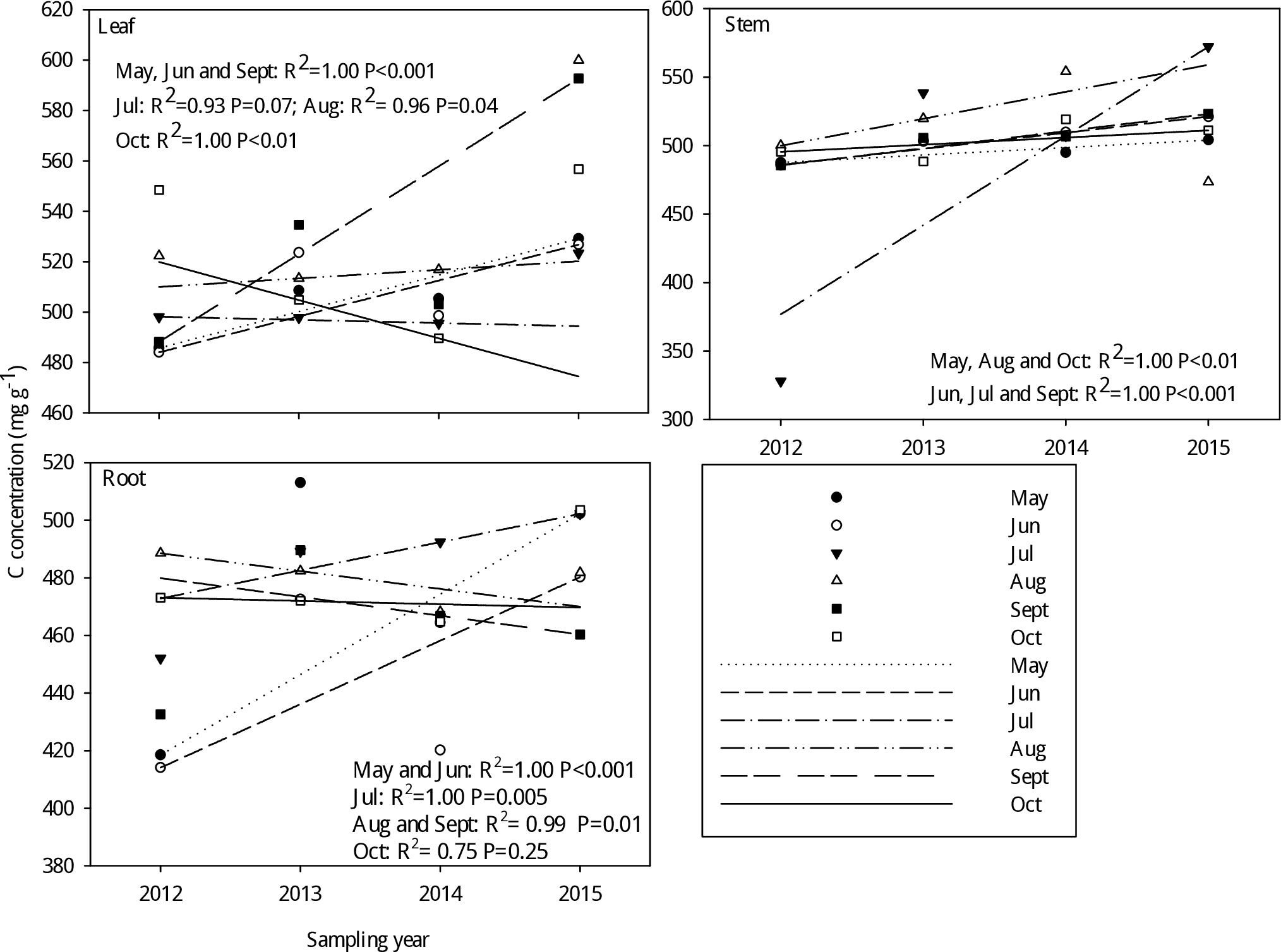

Supplement: S1 File — Line indicate that the liner regressions were significant at P <0.05. (ZIP) [file pone.0185163.s005.zip › S1_File/S2_Fig.tif]

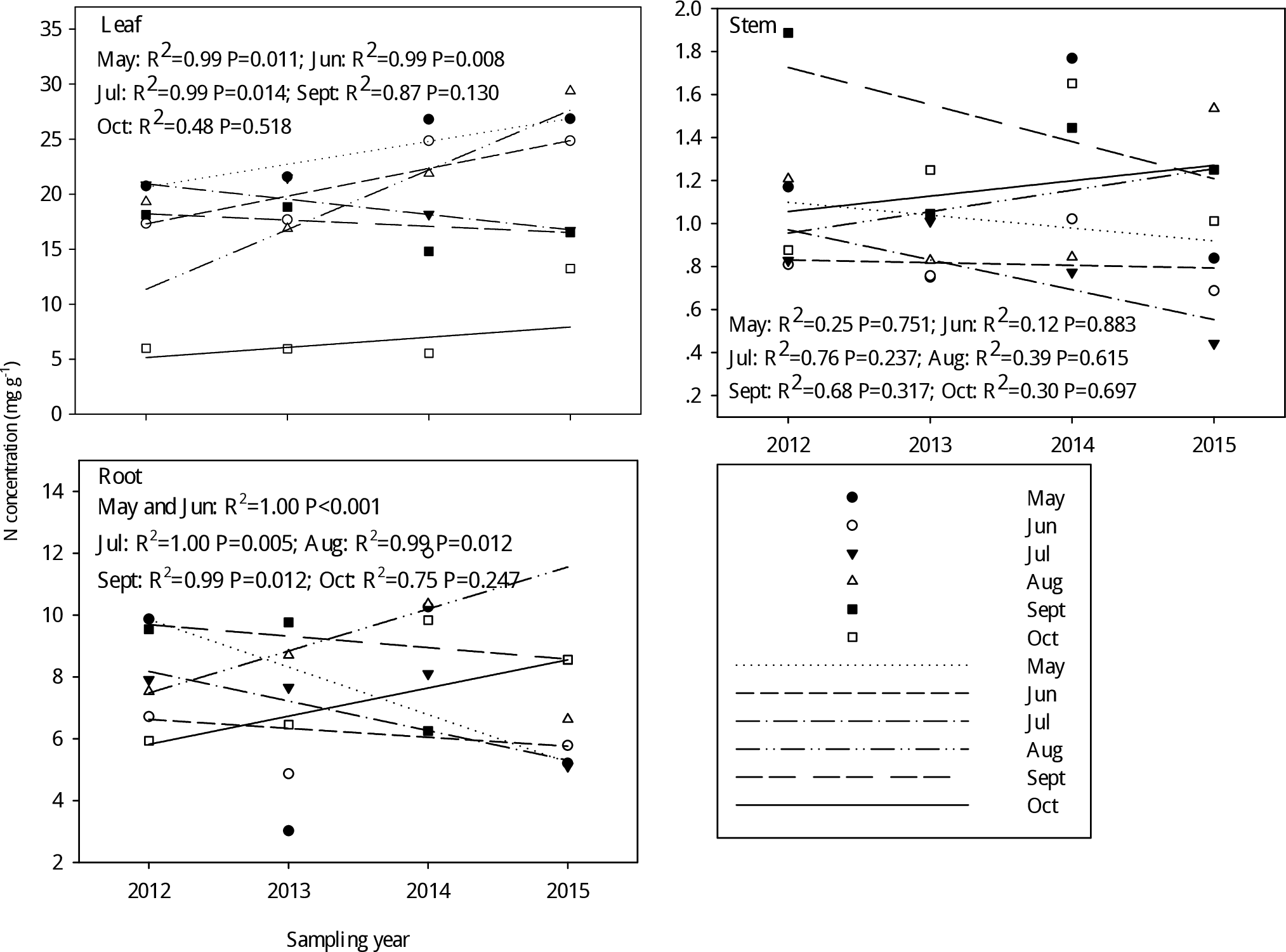

Supplement: S1 File — Line indicate that the liner regressions were significant at P <0.05. (ZIP) [file pone.0185163.s005.zip › S1_File/S3_Fig.tif]

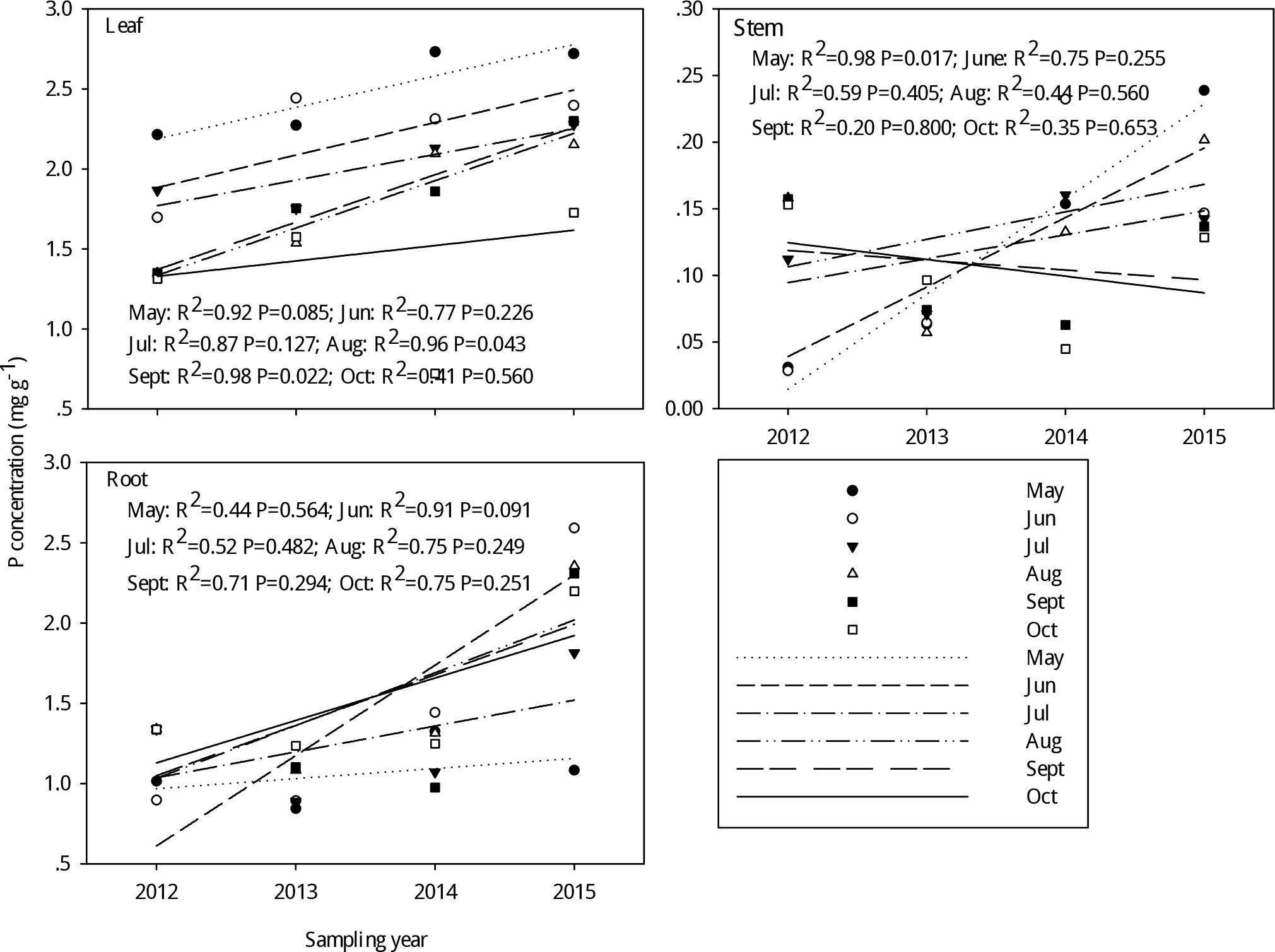

Supplement: S1 File — Line indicate that the liner regressions were significant at P <0.05. (ZIP) [file pone.0185163.s005.zip › S1_File/S4_Fig.tif]

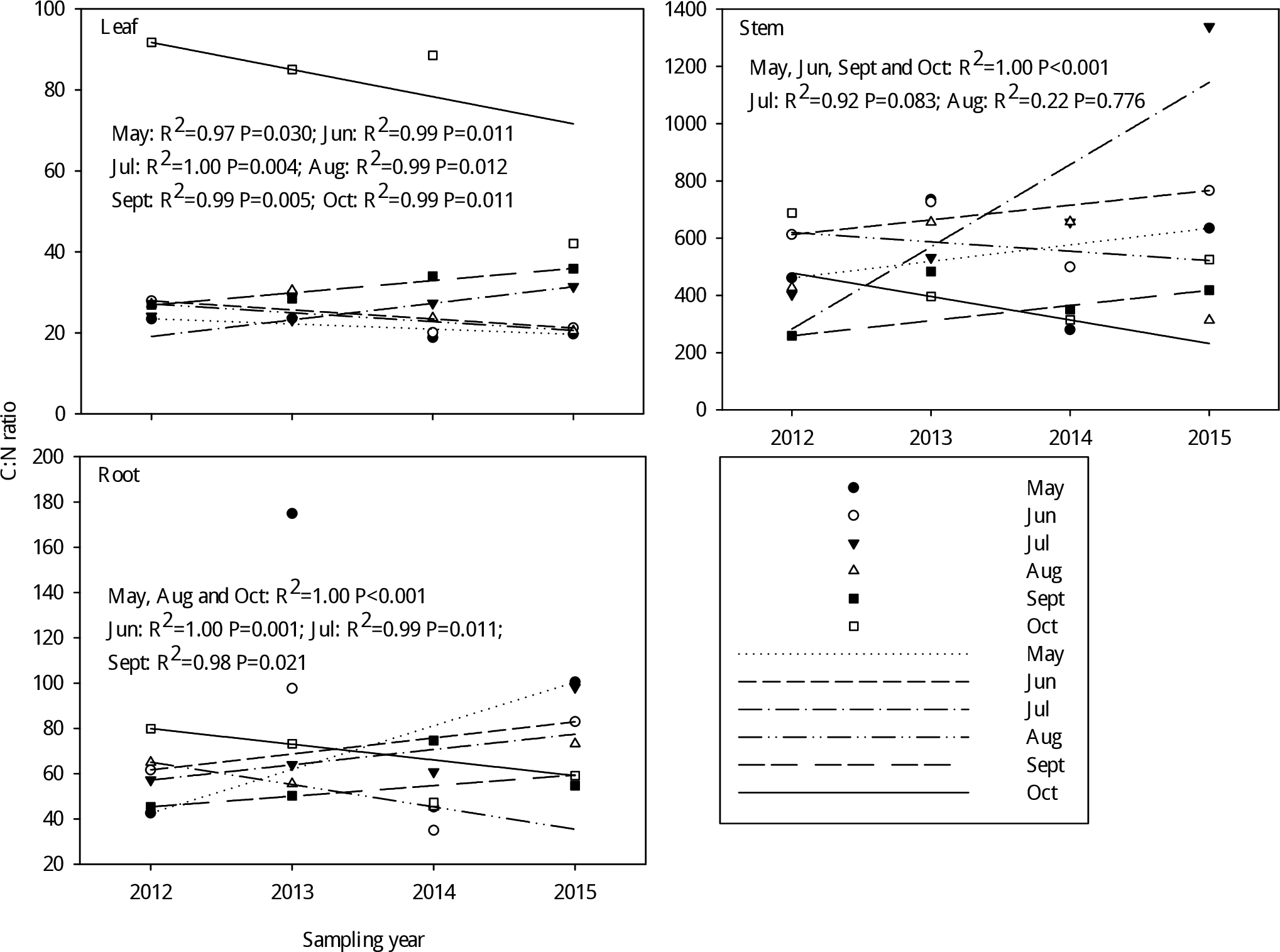

Supplement: S1 File — Line indicate that the liner regressions were significant at P <0.05. (ZIP) [file pone.0185163.s005.zip › S1_File/S5_Fig.tif]

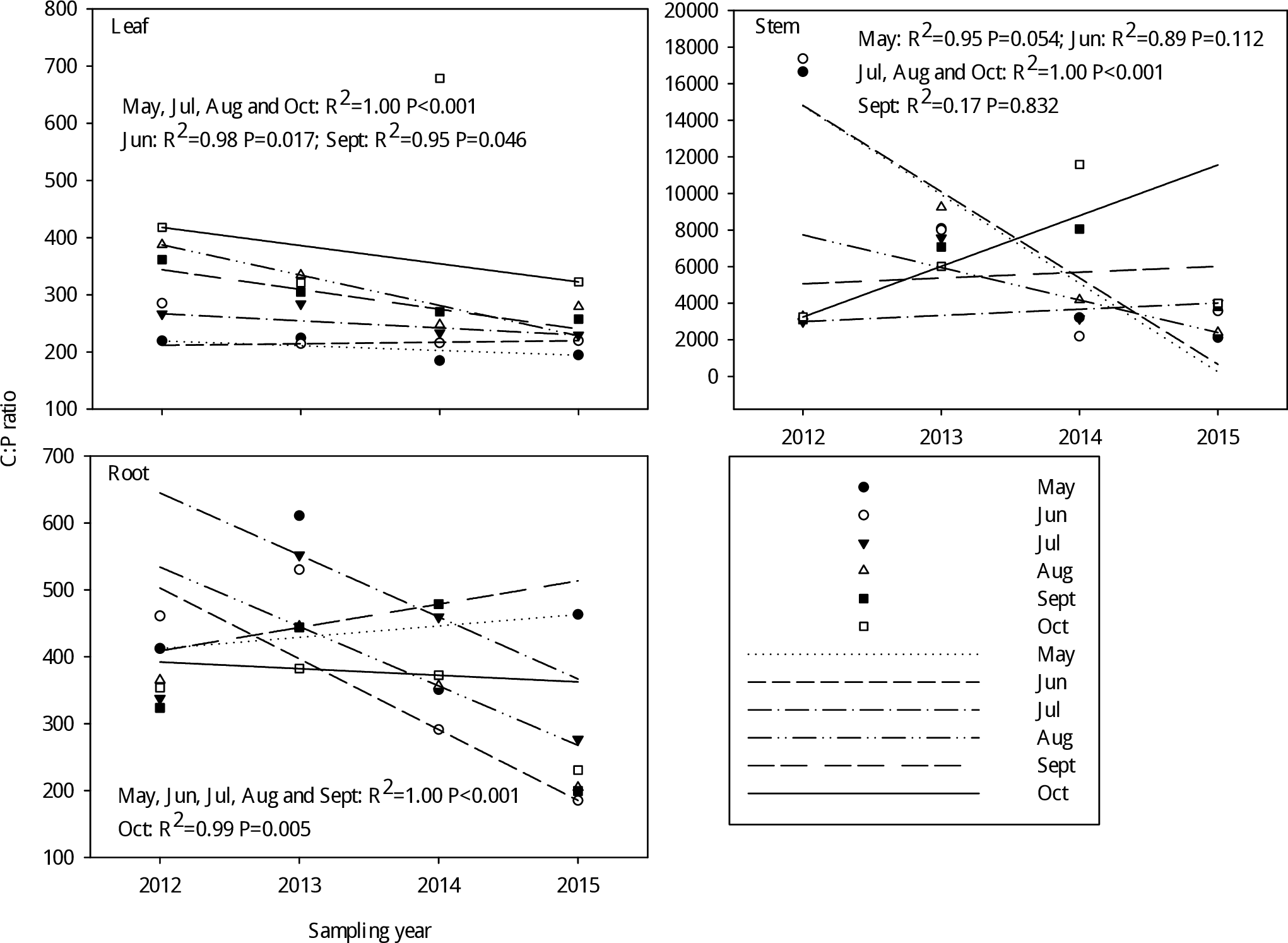

Supplement: S1 File — Line indicate that the liner regressions were significant at P <0.05. (ZIP) [file pone.0185163.s005.zip › S1_File/S6_Fig.tif]

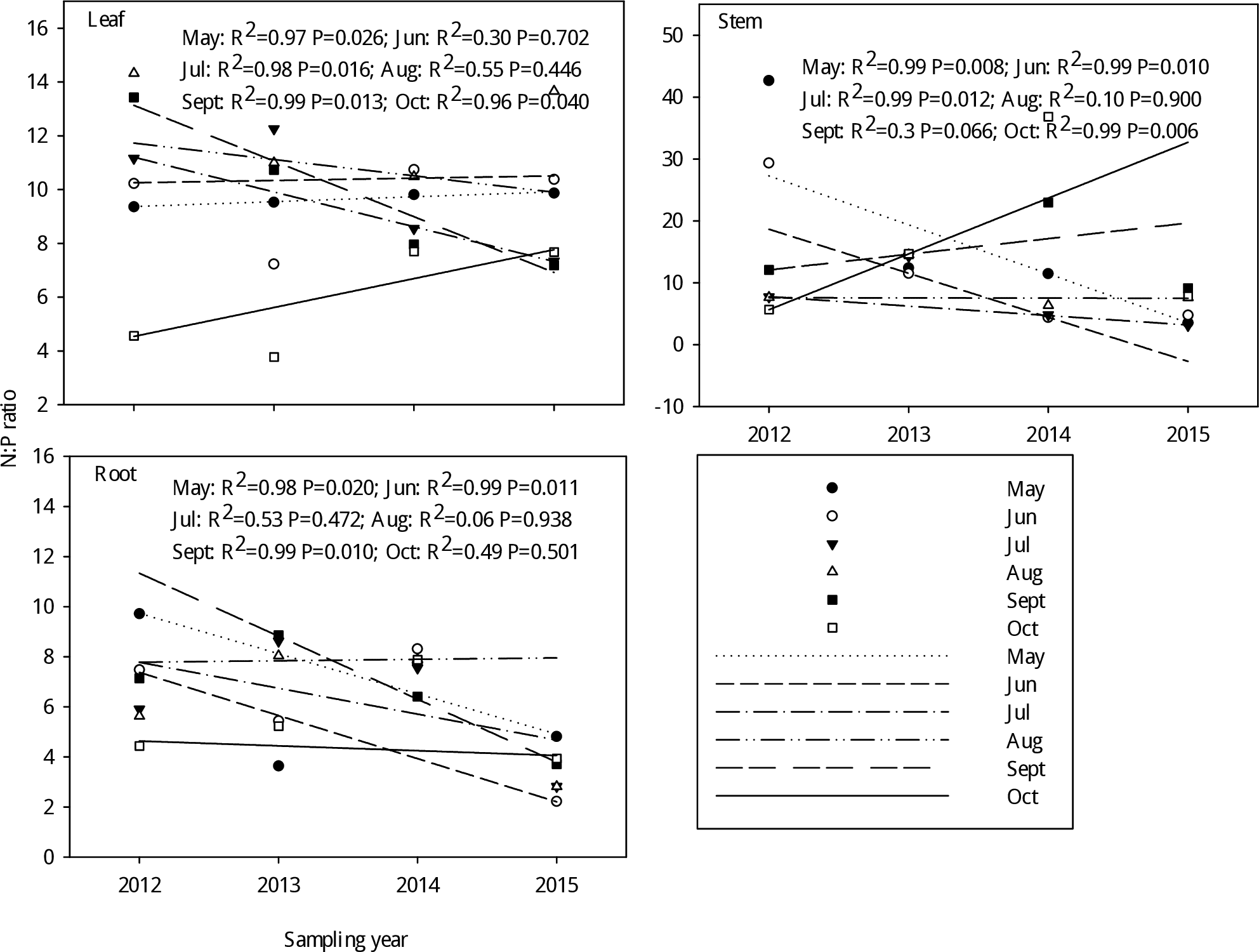

Supplement: S1 File — Line indicate that the liner regressions were significant at P <0.05. (ZIP) [file pone.0185163.s005.zip › S1_File/S7_Fig.tif]
