# Supplementary material for: Seasonal variations in carbon, nitrogen and phosphorus concentrations and C:N:P stoichiometry in different organs of a Larix principis-rupprechtii Mayr. plantation in the Qinling Mountains, China
Source: PLoS One. 2017 Sep 22;12(9):e0185163. doi: 10.1371/journal.pone.0185163 (PMC5609765; doi:10.1371/journal.pone.0185163)
Supplement: S2 File — (ZIP) [file pone.0185163.s006.zip › S2_File/S4_Table.docx]

**S4 Table. Analysis of variance for C, N and P concentrations of different organs of *L. principis-rupprechtii* Mayr. at various growing seasons from 2012-2015.**

| **Elment** | **Tissue** | **Analysis of variance at various growing seasons** | | | |  | **Elment** | **Year** | **Analysis of variance at various plant organs** | | | | | |
| --- | --- | --- | --- | --- | --- | --- | --- | --- | --- | --- | --- | --- | --- | --- |
|  |  |  | **Sampling time (df=5)** | | |  |  |  | **Sampling month (df=2)** | | | | | |
|  |  | **2012** | **2013** | **2014** | **2015** |  |  |  | **May** | **Jun** | **Jul** | **Aug** | **Sept** | **Oct** |
| **C** | **Leaf** | F=3.81 | F=3.95 | F=1.50 | F=25.07 |  | C | 2012 | F=3.53 | F=29.78 | F=56.17 | F=15.06 | F=8.72 | F=48.83 |
|  |  | P=0.027 | P=0.024 | P=0.269 | P=<0.001 |  |  |  | P=0.097 | P=0.001 | P=<0.001 | P=0.005 | P=0.017 | P=<0.001 |
|  | **Stem** | F=77.64 | F=2.60 | F=8.31 | F=6.39 |  |  | 2013 | F=0.23 | F=39.82 | F=3.43 | F=10.04 | F=11.27 | F=7.34 |
|  |  | P=<0.001 | P=0.082 | P=0.001 | P=0.004 |  |  |  | P=0.799 | P=<0.001 | P=0.101 | P=0.012 | P=0.009 | P=0.024 |
|  | **Root** | F=4.44 | F=3.58 | F=199.63 | F=3.45 |  |  | 2014 | F=29.99 | F=71.13 | F=1.11 | F=55.99 | F=130.42 | F=10.43 |
|  |  | P=0.016 | P=0.033 | P=<0.001 | P=0.037 |  |  |  | P=0.001 | P=<0.001 | P=0.390 | P=<0.001 | P=<0.001 | P=0.011 |
|  |  |  |  |  |  |  |  | 2015 | F=9.57 | F=5.36 | F=5.42 | F=39.01 | F=42.71 | F=74.93 |
|  |  |  |  |  |  |  |  |  | P=0.014 | P=0.046 | P=0.045 | P=<0.001 | P=<0.001 | P=<0.001 |
| **N** | **Leaf** | F=378.69 | F=345.66 | F=368.00 | F=166.40 |  | N | 2012 | F=116.72 | F=712.40 | F=614.57 | F=357.54 | F=858.07 | F=38.46 |
|  |  | P=<0.001 | P=<0.001 | P=<0.001 | P=<0.001 |  |  |  | P=<0.001 | P=<0.001 | P=<0.001 | P=<0.001 | P=<0.001 | P=<0.001 |
|  | **Stem** | F=3.35 | F=2.35 | F=441.22 | F=16.39 |  |  | 2013 | F=128.02 | F=158.08 | F=3687.49 | F=356.45 | F=1734.26 | F=290.54 |
|  |  | P=0.04 | P=0.105 | P=<0.001 | P=<0.001 |  |  |  | P=<0.001 | P=<0.001 | P=<0.001 | P=<0.001 | P=<0.001 | P=<0.001 |
|  | **Root** | F=51.76 | F=48.64 | F=126.67 | F=10.43 |  |  | 2014 | F=5091.49 | F=5152.91 | F=2918.67 | F=3773.07 | F=4609.73 | F=2414.40 |
|  |  | P=<0.001 | P=<0.001 | P=<0.001 | P=<0.001 |  |  |  | P=<0.001 | P=<0.001 | P=<0.001 | P=<0.001 | P=<0.001 | P=<0.001 |
|  |  |  |  |  |  |  |  | 2015 | F=194.88 | F=1182.52 | F=705.05 | F=718.36 | F=753.07 | F=259.47 |
|  |  |  |  |  |  |  |  |  | P=<0.001 | P=<0.001 | P=<0.001 | P=<0.001 | P=<0.001 | P=<0.001 |
| **P** | **Leaf** | F=3565.23 | F=68.91 | F=232.72 | F=183.80 |  | **P** | **2012** | F=608.40 | F=1490.91 | F=495.09 | F=282.19 | F=2379.82 | F=3317.86 |
|  |  | P=<0.001 | P=<0.001 | P=<0.001 | P=<0.001 |  |  |  | P=<0.001 | P=<0.001 | P=<0.001 | P=<0.001 | P=<0.001 | P=<0.001 |
|  | **Stem** | F=59.13 | F=1.01 | F=2006.75 | F=22.09 |  |  | 2013 | F=885.95 | F=776.74 | F=1808.24 | F=1412.23 | F=433.52 | F=80.80 |
|  |  | P=<0.001 | P=0.454 | P=<0.001 | P=<0.001 |  |  |  | P=<0.001 | P=<0.001 | P=<0.001 | P=<0.001 | P=<0.001 | P=<0.001 |
|  | **Root** | F=2386.76 | F=23.05 | F=114.45 | F=160.11 |  |  | 2014 | F=14617.48 | F=4199.11 | F=1684.45 | F=2540.30 | F=19118.76 | F=31159.49 |
|  |  | P=<0.001 | P=<0.001 | P=<0.001 | P=<0.001 |  |  |  | P=<0.001 | P=<0.001 | P=<0.001 | P=<0.001 | P=<0.001 | P=<0.001 |
|  |  |  |  |  |  |  |  | 2015 | F=3834.79 | F=1797.52 | F=5336.94 | F=705.92 | F=18742.11 | F=1379.41 |
|  |  |  |  |  |  |  |  |  | P=<0.001 | P=<0.001 | P=<0.001 | P=<0.001 | P=<0.001 | P=<0.001 |
