# Supplementary material for: Seasonal variations in carbon, nitrogen and phosphorus concentrations and C:N:P stoichiometry in different organs of a Larix principis-rupprechtii Mayr. plantation in the Qinling Mountains, China
Source: PLoS One. 2017 Sep 22;12(9):e0185163. doi: 10.1371/journal.pone.0185163 (PMC5609765; doi:10.1371/journal.pone.0185163)
Supplement: S2 File — (ZIP) [file pone.0185163.s006.zip › S2_File/S5_Table.docx]

**S5 Table. Analysis of variance for C:N, C:P and N:P ratios of different organs of *L. principis-rupprechtii* Mayr. at various growing seasons from 2012-2015.**

| **Elment** | **Tissue** | **Analysis of variance at various growing seasons** | | | |  | **Elment** | **Year** | **Analysis of variance at various plant organs** | | | | | |
| --- | --- | --- | --- | --- | --- | --- | --- | --- | --- | --- | --- | --- | --- | --- |
|  |  |  | **Sampling year (df=5)** | | |  |  |  | **Sampling month (df=2)** | | | | | |
|  |  | **2012** | **2013** | **2014** | **2015** |  |  |  | **May** | **Jun** | **Jul** | **Aug** | **Sept** | **Oct** |
| **C:N** | **Leaf** | F=139.80 | F=304.52 | F=277.77 | F=120.18 |  | **C:N** | **2012** | F=133.26 | F=537.39 | F=300.31 | F=341.84 | F=800.06 | F=39.91 |
|  |  | P=<0.001 | P=<0.001 | P=<0.001 | P=<0.001 |  |  |  | P=<0.001 | P=<0.001 | P=<0.001 | P=<0.001 | P=<0.001 | P=<0.001 |
|  | **Stem** | F=3.32 | F=2.52 | F=427.02 | F=16.68 |  |  | **2013** | F=141.04 | F=154.43 | F=1810.59 | F=304.83 | F=2434.40 | F=427.98 |
|  |  | P=0.041 | P=0.088 | P=<0.001 | P=<0.001 |  |  |  | P=<0.001 | P=<0.001 | P=<0.001 | P=<0.001 | P=<0.001 | P=<0.001 |
|  | **Root** | F=23.07 | F=47.06 | F=149.07 | F=9.10 |  |  | **2014** | F=4456.51 | F=4196.29 | F=2032.78 | F=8478.59 | F=5631.20 | F=1108.08 |
|  |  | P=<0.001 | P=<0.001 | P=<0.001 | P=0.001 |  |  |  | P=<0.001 | P=<0.001 | P=<0.001 | P=<0.001 | P=<0.001 | P=<0.001 |
|  |  |  |  |  |  |  |  | **2015** | F=169.40 | F=1204.47 | F=410.18 | F=363.56 | F=427.16 | F=257.89 |
|  |  |  |  |  |  |  |  |  | P=<0.001 | P=<0.001 | P=<0.001 | P=<0.001 | P=<0.001 | P=<0.001 |
| **C:P** | **Leaf** | F=78.72 | F=59.79 | F=166.86 | F=133.24 |  | **C:P** | **2012** | F=382.24 | F=1444.19 | F=351.13 | F=273.82 | F=1133.04 | F=4308.52 |
|  |  | P=<0.001 | P=<0.001 | P=<0.001 | P=<0.001 |  |  |  | P=<0.001 | P=<0.001 | P=<0.001 | P=<0.001 | P=<0.001 | P=<0.001 |
|  | **Stem** | F=54.27 | F=1.29 | F=692.44 | F=22.56 |  |  | **2013** | F=600.12 | F=780.12 | F=2655.98 | F=1260.71 | F=478.16 | F=90.22 |
|  |  | P=<0.001 | P=0.331 | P=<0.001 | P=<0.001 |  |  |  | P=<0.001 | P=<0.001 | P=<0.001 | P=<0.001 | P=<0.001 | P=<0.001 |
|  | **Root** | F=15.51 | F=20.59 | F=142.94 | F=128.58 |  |  | **2014** | F=18933.99 | F=2410.26 | F=1064.23 | F=2699.84 | F=21134.09 | F=13166.99 |
|  |  | P=<0.001 | P=<0.001 | P=<0.001 | P=<0.001 |  |  |  | P=<0.001 | P=<0.001 | P=<0.001 | P=<0.001 | P=<0.001 | P=<0.001 |
|  |  |  |  |  |  |  |  | **2015** | F=4341.69 | F=1228.17 | F=11430.01 | F=383.58 | F=9761.49 | F=1424.61 |
|  |  |  |  |  |  |  |  |  | P=<0.001 | P=<0.001 | P=<0.001 | P=<0.001 | P=<0.001 | P=<0.001 |
| **N:P** | **Leaf** | F=303.55 | F=194.78 | F=19.39 | F=53.38 |  | **N:P** | **2012** | F=11.53 | F43.45= | F=6.71 | F=500.80 | F=27.14 | F=0.25 |
|  |  | P=<0.001 | P=<0.001 | P=<0.001 | P=<0.001 |  |  |  | P=0.009 | P=<0.001 | P=<0.001 | P=<0.001 | P=0.001 | P=0.788 |
|  | **Stem** | F=11.48 | F=0.39 | F=1582.99 | F=13.77 |  |  | **2013** | F=8.99 | F=32.19 | F=61.29 | F=14.28 | F=8.12 | F=29.05 |
|  |  | P=<0.001 | P=0.85 | P=<0.001 | P=<0.001 |  |  |  | P=0.016 | P=0.001 | P=<0.001 | P=0.001 | P=0.02 | P=0.001 |
|  | **Root** | F=95.90 | F=22.70 | F=25.60 | F=10.78 |  |  | **2014** | F=77.30 | F=831.30 | F=128.74 | F=48.34 | F=1603.62 | F=1118.75 |
|  |  | P=<0.001 | P=<0.001 | P=<0.001 | P=<0.001 |  |  |  | P=<0.001 | P=<0.001 | P=<0.001 | P=<0.001 | P=<0.001 | P=<0.001 |
|  |  |  |  |  |  |  |  | **2015** | F=14.65 | F=74.52 | F=36.61 | F=704.34 | F=75.34 | F=24.82 |
|  |  |  |  |  |  |  |  |  | P=0.005 | P=<0.001 | P=<0.001 | P=<0.001 | P=<0.001 | P=0.001 |
